# Integrated *in vitro* and multi-cohort cross-omics analysis of HTLV-1-associated lung pathology reveals a RelA-dependent mechanism for monocyte recruitment and differentiation

**DOI:** 10.1186/s10020-026-01482-9

**Published:** 2026-04-23

**Authors:** Clément J. F. Heymann, Mieke Gouwy, Robin Hermans, Jean-Claude Twizere, Tatiane Assone, Jorge Casseb, Isaac Racine, Isabelle Cleynen, Edward L. Murphy, Roberta Bruhn, Dominique Schols, Evelien Vanderlinden, Johan Van Weyenbergh

**Affiliations:** 1 Laboratory of Molecular, Structural and Translational Virology, Department of Microbiology, Immunology and Transplantation, Rega Institute for Medical Research, KU Leuven, Leuven, Belgium; 2 Laboratory of Molecular Immunology, Department of Microbiology, Immunology and Transplantation, Rega Institute for Medical Research, KU Leuven, Leuven, Belgium; 3 Laboratory of Viral Interactomes Networks, Unit of Molecular and Computational Biology, Interdisciplinary Cluster for Applied Genoproteomics (GIGA Institute), University of Liège, Liège, Belgium; 4 Laboratory of Immunohematology and Forensic Hematology-LIM40, Department of Forensic Medicine, Medical Ethics, Social Medicine and Work, University of São Paulo Medical School, São Paulo, Brazil; 5 Laboratory for Complex Genetics, Department of Human Genetics, KU Leuven, Leuven, Belgium; 6 University of California San Francisco and Vitalant Research Institute, San Francisco, USA; 7 Vitalant Research Institute, San Francisco and Yale University, New Haven, USA; 8 Laboratory of Clinical and Epidemiological Virology, Department of Microbiology, Immunology and Transplantation, Rega Institute for Medical Research, KU Leuven, Leuven, Belgium

**Keywords:** HTLV-1, Transcriptomics, Lung, Inflammation, Monocytes, Bronchiectasis, GWAS, Interactome

## Abstract

**Background:**

Human T-lymphotropic virus type 1 (HTLV-1) infects up to ten million people worldwide and is associated with inflammatory diseases, including HTLV-1-associated myelopathy/tropical spastic paraparesis (HAM/TSP). Individuals with HAM/TSP are prone to pulmonary complications such as bronchiectasis, characterized by sustained mononuclear cell infiltration and elevated inflammatory mediators in bronchoalveolar lavage fluid. However, the epithelial mechanisms linking HTLV-1 exposure to lung inflammation remain poorly defined.

**Methods:**

To study epithelial signaling in response to HTLV-1 exposure, human alveolar epithelial A549 cells were co-cultured with HTLV-1-infected (MT-2 or MT-4 cells) T cells/supernatants or uninfected (Jurkat) T cells. Transcriptomic changes were assessed by RNA sequencing and pathway enrichment analyses, with key mediators validated by RT-qPCR. NF-κB dependency was evaluated using CRISPR/Cas9-mediated knockout of NF-κB RelA/p65. Functional consequences of epithelial activation were assessed using monocyte chemotaxis and differentiation assays in THP-1 cells and primary human monocytes. *In vivo* relevance was examined through integrative cross-omics analyses combining our own and publicly available bulk and single-cell transcriptomics, epigenomics, viral interactomics, and multi-ancestry genome-wide association studies (GWAS).

**Results:**

HTLV-1 exposure induced a robust epithelial antiviral and inflammatory transcriptional program in A549 cells, predominantly regulated by NF-κB signaling. Among the most strongly upregulated genes in A549 MT-2 co-cultures were the monocyte chemoattractant *MCP-1*/*CCL2* and the macrophage differentiation factor *CSF1*, as confirmed by RT-qPCR. CRISPR/Cas9-mediated knockout of NF-κB RelA/p65 demonstrated that CSF-1 induction is mechanistically dependent on NF-κB activation. Supernatants from HTLV-1-exposed epithelial cells promoted monocyte chemotaxis and macrophage differentiation in THP-1 cells and primary human monocytes. Transcriptomic data of people living with HTLV-1, HAM/TSP patients and idiopathic pulmonary fibrosis patients confirm *in vivo* expression of the *in vitro* gene signature, whereas single cell RNA-seq identified a unique myeloid subset in human lung, characterized by co-expression of *CCL2/ISG15/CXCL10.* Finally, GWAS analyses revealed ancestry-specific associations (*CCL2* for European and *CSF1* for African ancestry).

**Conclusions:**

We report an *in vitro* co-culture model that recapitulates HTLV-1-triggered lung inflammation through RelA/NF-kB-dependent release of pro-inflammatory cytokines and chemokines resulting in monocyte chemotaxis, activation and differentiation. This epithelial-myeloid inflammatory axis provides a relevant *in vitro* model that recapitulates *in vivo* HTLV-1-associated lung pathology.

**Supplementary Information:**

The online version contains supplementary material available at 10.1186/s10020-026-01482-9.

## Background

Human T-Lymphotropic virus type 1 (HTLV-1) is an enveloped, single-stranded RNA deltaretrovirus affecting up to ten million people worldwide (Einsiedel et al. 2021; Legrand et al. 2022). Mainly constrained to endemic areas, HTLV-1 infection is prevalent in the Southwestern part of Japan, sub-Saharan Africa and South America, the Caribbean Islands, and foci in Middle East and Australo-Melanesia Islands (Gessain and Cassar 2012). HTLV-1 has been defined as the principal causative agent of two severe diseases, Adult T cell leukemia/lymphoma (ATLL), an aggressive form of T-cell malignancy (Yoshida et al. 1984), and HTLV-1-associated myelopathy/tropical spastic paraparesis (HAM/TSP), an HTLV-1-induced neurologic disorder (Gessain et al. 1985). HTLV-1 infection can also induce acute inflammation-associated diseases, such as uveitis (Mochizuki et al. 1994; Nakao et al. 2018), Hashimoto’s thyroiditis (Kawai et al. 1992), and Graves' disease (Kamoi et al. 2022; Kawai et al. 1995). Finally, HTLV-1 carriers, mostly HAM/TSP patients, can exhibit pulmonary complications with the development of T-lymphocyte alveolitis, bronchiolitis or lymphocytic interstitial pneumonia (Einsiedel et al. 2021; Dias Á et al. 2022).

The first association between HTLV-1 and chronic respiratory disease, i.e. diffuse panbronchiolitis and idiopathic interstitial pneumonia, was published in 1986 (Kimura et al. 1986). This was followed by several reports of T-cell alveolitis and cases of lymphocytosis in broncho-alveolar lavage fluids from HAM/TSP patients (Couderc et al. 1988; Vernant et al. 1988; Sugimoto et al. 1987). Later, the lung was proven to contain one of the highest HTLV-1 proviral loads (PVL) compared to different organs obtained from the autopsy of an HAM/TSP patient (Sueyoshi et al. 1994). Currently, all clinical and pathological entities that result from HTLV-1-mediated inflammation of the lung are called HTLV-1-associated pulmonary disease (HAPD) (Einsiedel et al. 2021).

HAPD is frequently associated with the emergence of an inflammatory phenotype in the interstitium, airways, or alveoli (Einsiedel et al. 2021). Upon infection, respiratory cells produce pro-inflammatory cytokines and chemokines that recruit immune cells to the infected site (Teruya et al. 2008), where they can either suppress or facilitate viral dissemination. The extravasation of undifferentiated monocytes and peripheral macrophages plays a central role in regulating inflammation and disease progression.

Monocyte trafficking is primarily orchestrated through interactions between CC chemokine receptors (e.g., CCR2, CCR5) expressed on monocytes and their ligands (e.g., CCL2, CCL5) produced by inflamed tissues (Teruya et al. 2008; Shi and Pamer 2011; Ingersoll et al. 2011; Zargari et al. 2020). Once recruited, monocytes can differentiate into macrophages or dendritic cells under the influence of local growth factors, such as macrophage colony-stimulating factor (CSF-1) (Chen 2021; Yadav et al. 2023; Jaguin et al. 2013; Fleetwood et al. 2007). In the context of HTLV-1 infection, these differentiated monocyte-derived populations may act as viral reservoirs, sustaining viral persistence, and contributing to both immune regulation and tissue immunopathology (Souza et al. 2025; Castro-Amarante et al. 2015).

One important clinical manifestation of HAPD is bronchiectasis, a chronic lung disorder characterized by the irreversible dilatation and thickening of the walls of the airways (Steinfort et al. 2008). This respiratory disease has been repeatedly associated with HTLV-1 infection, particularly in individuals with HAM/TSP (Steinfort et al. 2008; Einsiedel et al. 2012; Honarbakhsh and Taylor 2015). The onset of bronchiectasis is linked to chronic inflammation in the lungs, which fosters the development of a fibrotic microenvironment within the affected tissues. In this setting, monocyte-derived alveolar macrophages have been implicated in the maintenance of pulmonary fibrosis, with their survival and activity supported by CSF-1/CSF-1R signaling pathways (Josh et al. 2020).

**Fig. 1:**
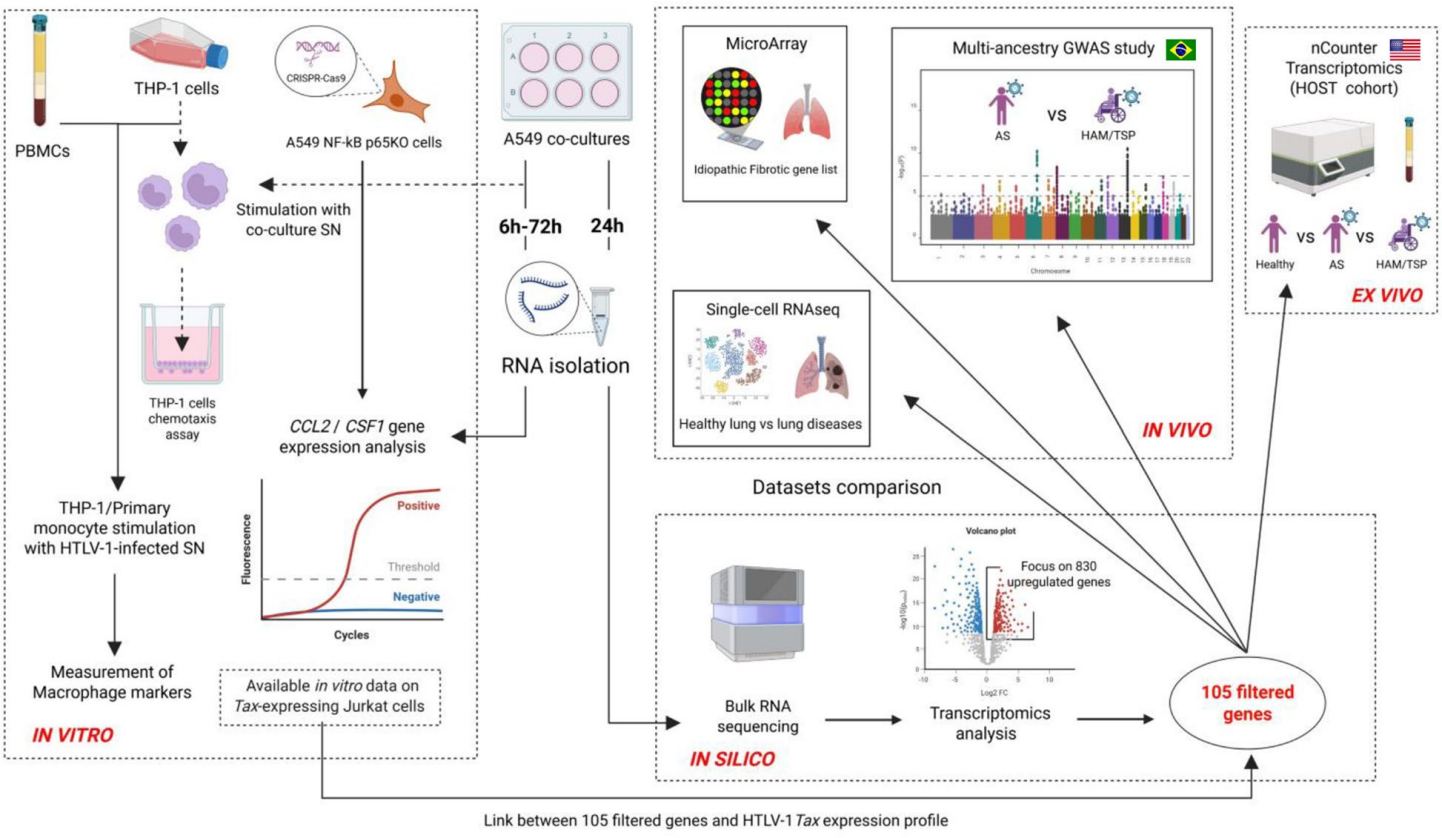
Overview of methodology and cohorts. The study employed an integrative approach to assess the effect of HTLV-1 infection on A549 lung epithelial cells. Differential gene expression analysis was conducted on *in vitro* co-culture samples. Findings were compared with available clinical datasets, highlighting an existing link between the onset of HTLV-1-associated HAM/TSP and idiopathic pulmonary fibrosis (IPF)

To better understand HTLV-1-associated inflammatory diseases, particularly HAPD in HAM/TSP patients, we used an integrated multi-omics framework. Our data-driven approach uncovers novel disease mechanisms and potential therapeutic targets for HTLV-1-associated lung pathology. We investigated transcriptome-wide responses triggered by HTLV-1 in A549 alveolar epithelial cells. As outlined in Figure 1, integrated multi-cohort multi-omics analyses, including bulk and single-cell transcriptomics, viral interactome data, and cross-ancestry genome-wide-association studies (GWAS), provide support for the *in vivo* relevance of these *in vitro* findings. Systems biology analysis showed RelA/NF-κB p65 as the major upstream transcription factor for lung-specific HTLV-1-upregulated genes. A central role for CSF-1-mediated recruitment and differentiation of monocytes was mechanistically linked to NF-κB activation, as demonstrated using a CRISPR/Cas9 A549 RelA knockout cell line.

## Methods

### Resources table

**Table:** 

**REAGENT OR RESOURCE**	**SOURCE**	**IDENTIFIER**
Antibodies		
Mouse anti-Human Clathrin	BD Biosciences	610500 (Western blot)
NF-kB p65	BD Biosciences	MAB5078 (Western blot)
Goat Anti-Mouse HRP	Agilent Dako	P0447 (Western blot)
Fc Block (Flow cytometry)	BD Biosciences	564220 (Flow cytometry)
Mouse anti-Human CD3 APC-Cy7	R&D Systems	557832 (Flow cytometry)
Mouse anti-Human CD14 BV421	R&D Systems	563743 (Flow cytometry)
Mouse anti-Human CD54 PE	BD Biosciences	347977 (Flow cytometry)
Bacterial and virus strains		
NEB 10-beta/Stable Competent* E.coli*	New England Biolabs	C3040H
Biological samples		
Buffy coats (Healthy donors)	Red Cross, Mechelen, Belgium	RKOV_19006
Chemicals, peptides, and recombinant proteins		
Recombinant Human Interleukin IL-1β	PeproTech	200-01B
Recombinant Human CCL2	PeproTech	3000-04
Recombinant Human Macrophage Colony-stimulating Factor	R&D Systems	216-MC-010
Critical commercial assays		
RNeasy Kit	Qiagen	74104
AllPrep DNA/RNA/Protein Kit	Qiagen	80004
High-Capacity cDNA Rever Transcription Kit	Applied Biosystems	4368814
GoTaq qPCR Master Mix	Promega	A6002
ATPlite Luminescence Assay System 96-well	Revvity	6016943
Human CCL2/MCP-1 ELISA kit	R&D Systems	DCP00
Human M-CSF/CSF-1 ELISA kit	R&D Systems	DMC00B
EasySep Human Monocyte Isolation Kit	STEMCELL Technologies	19359
Quick Ligation Kit	New England Biolabs	M2200S
Deposited data		
Transcriptomics data generated in this study were submitted to the GEO repository	National Institutes of Health (NIH)	GSE312068
Experimental models: Cell lines		
MT-2	NIH HIV Reagent Program	ARP237
MT-2 Tax shRNA	NIH HIV Reagent Program	ARP237 (Engineered)
MT-4	NIH HIV Reagent Program	ARP120
Jurkat	ATCC	TIB-152
THP-1	ATCC	TIB-202
A549	ATCC	CCL-185
A549 NF-kB p65 KO	ATCC	CCL-185 (Engineered)
HEK293T WT	ATCC	CRL-3216
Oligonucleotides		
Name	Sense strand	Antisense strand
CRISPR/Cas9 NF-kB *p65*KO Exon 4	TCAATGGCTACACAGGACCA	TGGTCCTGTGTAGCCATTGA
HTLV-1 *Tax* shRNA knockdown	GCAGATGACAATGACCATGA	TCATGGTCATTGTCATCTGC
* GAPDH*	TGATTTTGGAGGGATCTCGCTCCTGGAA	GTGAAGGTCGGAGTCAACGGATTTGGTCGT
* HBB* (β-Globin)	GCAAGAAAGTGCTCGGTG	CTACTCAGTGTGGCAAAGGTG
HTLV-1 *Tax*	CTACATCGTCACGCCCTACT	ATGAGTGATTGGCGGGGTAA
HTLV-1 *Hbz*	AGAACGCGACTCAACCGG	TGACACAGGCAAGCATCG
* IL1B*	AGATGATAAGCCCACTCTACAG	ACATTCAGCACAGGACTCTC
* TNF*	CCCGAGTGACAAGCCTGTAG	GATGGCAGAGAGGAGGTTGAC
* IL6*	ACAGCCACTCACCTCTTCAG	CCATCTTTTTCAGCCATCTTT
* CXCL8*	AGACAGCAGAGCACACAAGC	ATGGTTCCTTCCGGTGGT
* CSF1*	GTTTGTAGACCAGGAACAGTTGAA	CGCATGGTGTCCTCCATTAT
* IL34*	AATCCGTGTTGTCCCTCTTG	CAGCAGGAGCAGTACAGCAG
* CCL2*	GCCCCAGTCACCTGCTGTTAT	CTGCTTGGGGTCAGCACAGA
* ITGAM* (CD11b)	CAGCCTTTGACCTTATGTCATGG	CCTGTGCTGTAGTCGCACT
* CD14*	AGCCAAGGCAGTTTGAGTCC	TAAAGGACTGCCAGCCAAGC
* FCGR3A* (CD16)	ATGTGTCTTCAGAGACTGTGAAC	TTTATGGTCCTTCCAGTCTCTTG
* CD36*	GCCAAGGAAAATGTAACCCAGG	GCCTCTGTTCCAACTGATAGTGA
* CD68*	GCTACATGGCGGTGGAGTACAA	ATGATGAGAGGCAGCAAGATGG
* CD86*	CTGCTCATCTATACACGGTTACC	GGAAACGTCGTACAGTTCTGTG
* CD163*	CAGGAAACCAGTCCCAAACA	AGCGACCTCCTCCATTTACC
* SIGLEC1* (CD169)	CCTCGGGGAGGAACATCCTT	AGGCGTACCCCATCCTTGA
* MRC1* (CD206)	TTCGGACACCCATCGGAATTT	CACAAGCGCTGCGTGGAT
Recombinant DNA		
pPLentiCRISPRv2 plasmid	Addgene	52961
pCMV-VSV-G	Addgene	8454
pLV-SmCherry	Addgene	36084
pLKO.1	Addgene	10878
psPAX2	Addgene	12260
pMD2.G	Addgene	12259
Software and algorithms		
CLC Main WorkBench	Qiagen	v22.0.2
Enrichr	Icanh School of Medicine at Mount Sinai (Ma’ayan Laboratory)	https://maayanlab.cloud/Enrichr/cha
ShinyGO	South Dakota State University	v0.85
FlowJo	BD Biosciences	v10.8.1
GraphPad Prism	GraphPad Software	v10.6.0
Design and Analysis Software	ThermoFisher Scientific	v2.6.0
Image Lab	Bio-Rad	v6.1
R	The R Project for Statistical Computing	v4.4.2
RStudio	Posit PBC	V2024.09.01
STRING	Global Biodata Coalition and Elixir	v12.0
CELLxGENE Census	Chan Zuckerberg Initiative	CZ CELLxGENE Discover - Cellular Visualization Tool
CIBERSORTx	Stanford University	(Newman et al. 2019).

## Method details

### Reagents

Recombinant human interleukin-1 beta (IL-1β) (#200-01B) and recombinant human CCL2 (#300-04) were purchased from PeproTech (Cranbury, NJ, USA). Recombinant Human Macrophage Colony-stimulating Factor (M-CSF) protein (#216-MC-010) was obtained from R&D Systems (Minneapolis, MN, USA). Mitomycin C (#A11491) was acquired from Adooq Bioscience (Irivine, CA; USA).

### Plasmids

pLentiCRISPRv2 plasmid was a gift from Feng Zhang (Addgene, Watertown, MA, USA, #52961). pCMV-VSV-G was a gift from Bob Weinberg (Addgene, #8454). LentiCRISPRv2 constructs were made based on the Zhang Laboratory protocol, using Quick ligase (New England Biolabs, Ipswich, MA, USA, #M2200S). pLV-SmCherry control plasmid was a gift from Pantelis Tsoulfas (Addgene, #36084). Concerning the design of shRNA cell lines, pLKO.1 - TRC cloning vector was a gift from David Root (Addgene, #10878). psPAX2 (Addgene, #12260) and pMD2.G (Addgene, #12259) were gifts from Didier Trono.

### Cell cultures

#### Cell lines

Human lung carcinoma A549 (#CCL-185), Jurkat (#TIB-152) and THP-1 cells (#TIB-202) were purchased from American Type Culture Collection (ATCC, Manassas, VA, USA). HEK293T cells were received from Prof. Jason Moffat (Donnelly Centre, University of Toronto, Toronto, ON, Canada). MT-2 (active HTLV-1 producing cells) (#ARP237) and MT-4 (latently infected with HTLV-1) (#ARP120) cells were purchased from the National Institutes of Health (NIH) HIV Reagent Program.

A549 cells were maintained in HAM’s F-12K medium (Thermo Fisher Scientific [TFS], Waltham, MA, USA) supplemented with 5% fetal bovine serum (FBS, Cytiva, Marlborough, MA, USA) and 2mM L-Glutamine (TFS). HEK293T cells were grown in DMEM supplemented with 10% FBS and 2mM L-Glutamine (TFS). Jurkat, THP-1, MT-2 and MT-4 cells were cultured in RPMI (TFS) supplemented with 10% FBS (Cytiva) and 2mM L-Glutamine (TFS).

#### Isolation and purity assessment of monocytes

Monocytes were isolated from buffy coats of healthy donors (Red Cross, Mechelen, Belgium; contract No. RKOV_19006) with informed consent. Erythrocytes were removed using HetaSep (STEMCELL Technologies, Vancouver, BC, Canada, #07906) and human peripheral blood mononuclear cells (PBMCs) were obtained via density gradient centrifugation over Lymphoprep (STEMCELL Technologies, #18061). PBMCs were rotated overnight at 4 °C to promote monocyte aggregations. Monocyte isolation was performed using the EasySep Human Monocyte Isolation Kit (STEMCELL Technologies, #19359) according to the manufacturer's protocol. PBMCs (2x10⁸ cells in 2 mL EasySep Buffer) were incubated with 100 µL each of Isolation Cocktail and Platelet Removal Cocktail for 5 min at room temperature (RT), followed by addition of 100 µL of Magnetic Beads and an additional 5 min incubation. The volume was adjusted to 2.5mL, and negative selection was performed using the EasySep Magnet to collect untouched CD14⁺ monocytes.

For purity assessment, PBMCs and isolated monocytes were washed and resuspended in PBS with 2% FBS at 10×10⁶ cells/mL. Human BD Fc Block (BD Biosciences, San Jose, CA, USA, #564220) was added (25 µg per sample), and cells were incubated for 20 min at RT. Cells were then stained at 2×10^5^ cells/mL in 100 µL PBS + 2% FBS with 2.5 µL of each selected antibody. Staining was performed using anti-human CD3 APC-Cy7 (R&D System, #557832) and anti-human CD14 BV421 (R&D System, #563743), both from BD Biosciences. After 1 h at 4 °C, cells were washed with PBS + 2% FBS and fixed in 200 µL PBS + 2% PFA.

#### Genome editing

A CRISPR/Cas9-mediated RelA/NF-kB p65 knockout pool A549 cell line was generated using designed sgRNA sequences (Resources Table). Guide sequences were cloned into the pLentiCRISPRv2 plasmid (Addgene, #52961), according to the standard cloning protocol. For lentiviral particle production, HEK293T cells were plated in 40 mL supplemented DMEM in T150 (TPP, Trasadingen, Switzerland) flasks at 45% confluency and incubated overnight. One hour prior to transfection using the Lipofectamine LTX and Plus Reagent (TFS, #15338100), DMEM medium was removed and 13 mL OptiMEM (TFS, #31985062) was added to the flasks. The transfection mix was made by diluting 200 μl of PlusTM Reagent (TFS, #15338100) in 4 mL of OptiMEM, in addition to 20 µg transfer plasmid (either lentiCRISPR v2 containing the sgRNAs, or pLV-mCherry), 10 µg of envelope vector pCMV-VSV-G (env gene) and 15 µg of packaging vector psPAX2 (gag, pol, rev and tat genes). In addition, 100 µl of lipofectamine LTX (TFS, #15338100) was diluted in 4 mL OptiMEM and added to the DNA and PlusReagent mix after 5 min. After 20 min of incubation at RT, the mixture was added in a dropwise manner to the T-150 flask HEK293T cells in OptiMEM. Six hours after transfection, the medium was removed and replaced with 30 mL DMEM containing 1% BSA. The supernatant containing lentiviral particles was harvested 60 h after transfection and stored at −80°C. A549 target cells were transduced with lentiviruses expressing a pool of the 2 sgRNAs and then selected with puromycin (1.5 mg/mL) for 3 days. A similar approach was followed to generate a MT-2 Tax shRNA cell line. Of note, packaging of shRNA lentiviruses was performed using psPAX2 and pMD2.G as envelop plasmids.

#### Validations of CRISPR/Cas9-mediated RelA/NF-kB p65 knockout pool A549

a Direct confirmation by Western blot

A549 cells were seeded at 4×10^5^ cells/well in 6-well plate. After 24 h, cells were washed with ice-cold PBS and lysed on ice in Nonidet P-40 buffer (NP-40, TFS, #AAJ19628AP), supplemented with 50 mM Tris HCI, pH 8.0, 150 mM NaCl, protease inhibitor cocktail (Roche, Basel, Switzerland, #4693132001) and phenylmethanesulfonyl fluoride (Merck, Darmstadt, Germany, #P7626). Cellular debris were removed by centrifuging lysates at 17.000 x g for 10 min at 4°C. Extracted proteins were quantified using the Pierce BCA Protein assay kit (Cell Signaling Technology, Danvers, MA, USA, #7780S). After being incubated for 10 min at 95 °C in reducing buffer (120 mM Tris-HCl, pH 6.8, 4% SDS, 20% glycerol, 100 mM dithiothreitol (Merck) and 0.02% bromophenol blue), lysates were run on a 4–12% Bis-Tris gel (Bio-rad, Hercules, CA, USA, #3450124) and blotted on a nitrocellulose membrane (Bio-Rad) using the Trans-Blot Turbo system (Bio-Rad). Samples were blocked in 5% non-fat milk in TBST before incubating overnight at 4 °C with Mouse anti-Human NF-kB p65 (BD Biosciences, #MAB5078) or Mouse anti-Human Clathrin antibody (BD Biosciences, #610500). After thorough washing in TBST, blots were incubated for 1 h in HRP-conjugated Goat anti-Mouse antibody (Agilent Dako, Santa Clara, CA, USA, #P0447). After a final washing step, bands were detected using SuperSignal West Femto chemiluminescence reagent (TFS, #P134095) and analyzed with a ChemiDocMP (Bio-Rad).

b Indirect confirmation by flow cytometry (ICAM-1 staining)

A549 control and A549 NF-kB p65 KO were seeded at 4×10^5^ cells per well in 6-well plates and incubated at 37 °C overnight. The next day, the cells were stimulated for 6 h with 50 ng/mL TNF. After incubation, the cells were washed and resuspended in 50 mL PBS supplemented with 2% FBS. The cells were then incubated with a PE Mouse anti-Human CD54 antibody (BD Biosciences, #347977) for 30 min at 4°C. The cells were then washed once in PBS + 2% FBS and fixed in 2% PFA diluted in PBS.

### Bulk RNA sequencing

#### Sample preparation

A549 cells were seeded at 4×10^5^ cells per well in 6-well plates 24 h prior to infection or stimulation with cell culture supernatant (SN). On day 1, Jurkat, MT-4, and MT-2 cells were resuspended in RPMI at 4×10^5^ cells per ml and treated with 5 µM mitomycin C (Adooq Bioscience,#A11491) for 20 min at 37 C. Cells were washed with HAM’s F-12K medium and resuspended in the same medium at 4×10^5^ cells per ml. Finally, Jurkat, MT-4 or MT-2 cells were co-cultured with A549 cells at a final ratio of 1:1 (A549:Jurkat, MT-4 or MT-2). In parallel, SN from MT-4 and MT-2 cultures (collected 3 days post-passage) were filtered through a 0.45 µm filter (Corning, NY, USA, #431220) and used to stimulate A549 cells (mixed with control medium at a 1:1 ratio). After 24 h at 37 C, A549 cells were washed with PBS to remove non-adherent cells, detached with 0.25% trypsin, and incubated with CD25 Dynabeads (Invitrogen, TFS, #11157D) for negative isolation of A549 cells, according to the manufacturer’s instructions. RNA was then extracted using the RNeasy Mini Kit (Qiagen, Venlo, the Netherlands #74104) and the samples were submitted to the Genomics core facility (KU Leuven, Belgium) for RNA sequencing analysis (Supplementary Table 1).

#### Principal component analysis

Principal Component Analysis (PCA) was performed to reduce the dimensionality of the dataset and to identify patterns in the multivariate data. The analysis was conducted using the prcomp function in R (v4.4.2).

#### CIBERSORTx deconvolution analysis

To assess potential contamination of A549 transcriptomes with residual HTLV-1-infected donor cell material (MT-2 or MT-4), digital cytometry was performed using CIBERSORTx (Newman et al. 2019). Normalized RNA-seq counts obtained from the different A549 co-cultures were input in CIBERSORTx for deconvolution. A custom signature matrix was generated from bulk RNA-seq profiles of MT-2 and MT-4 cells, derived from the same variants used in our *in silico* omics study (Vanderlinden *et al*., unpublished data). A549 monoculture from our RNA-seq analysis was used to define the epithelial cell profile in the signature matrix. The analysis was run in absolute mode with 100 permutations to estimate the relative abundance of MT-2 and MT-4-derived transcripts in each A549 sample. The resulting cell fraction estimates were statistically compared across experimental conditions using one-way ANOVA, followed by Dunnett’s multiple comparisons test to evaluate significant increases in donor cell-associated transcript signatures relative to controls. All values in both signature and mixture matrixes were presented as log(2) values.

#### Differential gene expression analysis

To evaluate the impact of HTLV-1 infection on the A549 transcriptome, differential gene expression analysis was performed on various A549 co-cultures (Supplementary Tables 2-6). In this model, the A549-Jurkat co-culture transcriptome served as control to identify potential gene expression changes. Fold changes were also compared to established alveolar lung epithelial cell markers from single-cell data to focus on A549-specific effects (see 4.7). Raw reads were quality-checked with FastQC (Newman et al. 2019) (v0.11.7), adapters trimmed using Trimmomatic (Bolger et al. 2014) (v0.39) and aligned to the hg38 genome and transcriptome using hisat (Kim et al. 2019) with default settings. Gene counts were obtained via FeatureCounts (Subread package (Liao et al. 2019)), and differential expression analysis was done with DESeq2 (Love et al. 2014) in R software (v4.4.2). *P*-values were adjusted using the Benjamini-Hochberg method to control FDR. Simultaneously, all obtained mRNA reads were realigned and mapped to the reference HTLV-1 genome (J02029.1) to detect viral reads within the total RNA-seq data.

#### Kyoto Encyclopedia of Genes and Genomes / Gene Ontology enrichment analyses

Kyoto Encyclopedia of Genes and Genomes (KEGG) enrichment (Kanehisa et al. 2017) and Gene Ontology (Alterovitz et al. 2007) (GO) analyses were performed on the identified differentially expressed genes using the clusterProfiler (Yu et al. 2012), org.Hs.eg.db (v3.19.0), enrichplot (v1.28.4), and ggplot2 (Wickham 2016) packages in R software (v4.4.2). Bar plots displaying the 40 most significantly enriched pathways for upregulated genes in A549 cells co-cultured with MT-2 cells were generated. From these, 18 pathways were selected based on their clinical relevance regarding HTLV-1 infection, to derive a filtered gene list of 105 upregulated genes in A549-MT-2 co-cultures for downstream protein-level analysis. Focus was given to pathways associated with oncogenic, neuroinflammatory, and respiratory viral infections.

In parallel, dot plots of significantly enriched GO terms were created to visualize enrichment results across five Gene Ontology (GO) categories: (1) viral infection, (2) inflammation, (3) NF-κB activation, (4) cell chemotaxis, and (5) cell differentiation.

#### Protein-protein interaction network

A protein–protein interaction (PPI) network was generated using the filtered list of 105 upregulated genes identified from A549–MT-2 co-culture transcriptomic data. Interactions were retrieved from the STRING database (Szklarczyk et al. 2019) (v11.5) with a maximum confidence score of 0.9. The resulting PPI network was used to investigate signaling pathways and potential functional associations among the identified proteins. Particular attention was given to pathways directly linked to HTLV-1 infection (hsa05166), as well as those connecting infection to clinical outcomes such as bronchiectasis (HP:0002110) and increased levels of tissue monocytes (BTO:0008876). Additionally, the analysis emphasized monocyte responses within the pulmonary microenvironment by assessing enrichment in Gene Ontology (GO) terms related to monocyte chemotaxis (GO:0002548) and differentiation (GO:0045655). All selected pathways were significantly enriched, with a padj <0.05, after stringent FDR correction.

#### Upstream transcription factor enrichment and NF-κB RelA/p65 ChIP-seq data analyses

Prior to any in vitro experiments, an upstream transcription factor (TFs) enrichment analysis was performed to identify TFs likely to regulate the 105 pre-selected genes. This analysis was carried out using the open-source platform Enrichr (Kuleshov et al. 2016). Multiple databases documenting TF activity across diverse gene sets were assessed, with the TRRUST (Han et al. 2018) and ENCODE (Consortium, E.P. 2012) databases ultimately chosen for the final analysis (Supplementary Table 7, Supplementary Table 8). In parallel, the filtered KEGG list of 105 genes was compared against the ARCHS4 Tissue database (Lachmann et al. 2018) to determine the most likely tissue targets associated with enriched TFs.

Publicly available RelA ChIP-seq datasets generated from BEAS-2B airway epithelial cells were obtained from a previously published study (GSE79803) (Kadiyala et al. 2016). The analysis focused on characterizing genome-wide binding patterns of the NF-κB transcription factor RelA/p65 at key inflammatory gene loci in BEAS-2B cells under control condition or after stimulation with the pro-inflammatory cytokine TNF for 1 h (20 ng/ml).

#### Cohort presentation and data collection

Transcriptomics data were first compared with publicly available transcriptomes from whole blood samples (Tattermusch et al. 2012). Subsequently, the results were contrasted with recent findings from multi-ancestry Genome-Wide Association Study (GWAS) data (Van Weyenbergh et al. 2025). Finally, both the transcriptomics and GWAS data were compared with an additional *ex vivo* HAM/TSP dataset (HOST cohort) (Kwaan et al. 2006; Assone et al. 2022), as well as with a curated Idiopathic Pulmonary Fibrosis (IPF) gene list, to evaluate potential links between HTLV-1–induced inflammation and clinical outcomes. All cohorts are summarized in Table 1 and described in detail in the referenced publications (Tattermusch et al. 2012; Van Weyenbergh et al. 2025; Kwaan et al. 2006; Assone et al. 2022).

**Table 1:** Summary of cohorts included in the omics analysis

**Datasets used for Idiopathic Pulmonary Fibrosis (IPF) gene list**				
**Cohort**	**Number Control patients**	**Number IPF Patients**	**Sample Type**	**Omics Platform**
GSE32537	50	167	RNA (lung)	MicroArray
GSE47460	108	254	RNA (lung)	MicroArray
GSE53845	8	40	RNA (lung)	MicroArray
GSE70866	20	212	RNA (lung)	MicroArray
GSE110147	11	22	RNA (lung)	MicroArray

**Whole blood HTLV-1 transcriptomic signature**				
**Cohort**	**Number Control patients**	**Number AS/HAM patients**^**a**^	**Sample Type**	**Omics Platform**
GSE29312	9	20/10	RNA (Blood)	MicroArray

**Cohort Genome-wide associated study**				
**Cohort**	**Number AS patients**^**a**^	**Number HAM patients**^**a**^	**Sample Type**	**Omics Platform**
Brazil	535	416	DNA	SNP Array

**UCSF Cohort (nCounter)**				
**Cohort**	**Number Control patients**	**Number AS/HAM patients **^**a**^	**Sample Type**	**Omics Platform**
HOST (USA)	4	4/4	Blood	nCounter

^a^*AS* Asymptomatic, *HAM *HTLV-1-associated myelopathy/tropical spastic paraparesis

Our study relied on publicly available data from the Gene Expression Omnibus (GEO), ensuring no ethical concerns or conflicts of interest. All patient data in the GEO datasets were previously collected under ethical approval and can be freely accessed and analyzed in accordance with GEO’s usage policies.

##### Epithelial cell status

To validate the epithelial identity of A549 cells across co-culture conditions, RNA-seq–identified genes were compared against a curated list of epithelial cell markers sourced from the Panglao database (Franzén et al. 2019). Additionally, a correlation analysis was conducted by comparing raw gene counts from our RNA-seq data with aggregated read counts from multiple A549 RNA-seq experiments available in the ARCHS4 database (Lachmann et al. 2018). Prior to comparison, the data were filtered to retain genes with a minimum of 200 reads in at least three samples to only look at commonly expressed genes in the defined cell line. Genes common to both datasets were then used to compute Pearson correlation coefficients, and a correlation heatmap was generated to assess sample similarity.

##### Idiopathic Pulmonary fibrosis (IPF)

To pinpoint IPF-related markers among differentially expressed genes (DEGs), a filtered list of IPF-associated genes was compiled from 5 GEO Series Matrix Files (GSE32537 (Yang et al. 2013), GSE47460 (Peng et al. 2016; Anathy et al. 2018; Kim et al. 2015; Yu et al. 2018; Tan et al. 2016), GSE53845 (DePianto et al. 2015), GSE70866 (Prasse et al. 2019), and GSE110147 (Cecchini et al. 2018)), including healthy and IPF patient samples. Expression data were normalized and analyzed with the limma package (Ritchie et al. 2015) (v.4.4.2).

##### HTLV-1 gene signature in the human lung cell atlas

To contextualize the results in a clinical perspective, the gene list derived from KEGG enrichment analysis was further examined using the Human Lung Cell Atlas (HLCA) database (Sikkema et al. 2023), accessed via CellxGene Census. Relative gene expression levels were assessed using lung single-cell RNA-seq data. The HTLV-1 gene signature was compared across both healthy lung tissues and tissues affected by inflammatory lung diseases, including idiopathic pulmonary fibrosis (IPF), COVID-19, hypersensitivity pneumonitis, and pulmonary sarcoidosis. Furthermore, CCL2 expression in the lung was analyzed alongside ISG15 and CXCL10 within a defined myeloid cell subset.

### RT-qPCR

A549 cells were seeded at 4×10^5^ cells per well in 6-well plates 24 h before infection or stimulation with cell culture SN. Unless specified differently, A549 cells were co-cultured with mitomycin-treated Jurkat, MT-4, or MT-2 cells (or SN) at a 1:1 ratio for 48 h. For the kinetics experiments, co-cultures were incubated for 6 h, 24 h, 48 h and 72 h. Then, the A549 cells were washed, and total RNA was extracted using the RNeasy Kit (Qiagen, #74104), according to the manufacturer’s instruction. First-strand cDNA was synthesized from 350 ng RNA using the High-Capacity cDNA Reverse Transcription Kit (Applied Biosystems, #4368814) and 10-fold diluted. Then, qPCR was performed with GoTaq qPCR Master Mix (Promega, Madison, WI, USA; #A6002) to quantify changes in mRNA expression levels. All primers (Resources Table) were used at a final concentration of 500 nM. Amplification was performed on a QuantStudio 5 Real-Time PCR System (TFS), and consisted of a 2-min initial activation at 95 °C, followed by 40 thermal cycles of 15 s at 95 °C and 60 s at 60°C. A dissociation profile was taken at the end to confirm the specificity of the PCR amplification. Relative changes in gene expression were determined using the DDC_t_ values obtained for all tested primer pairs and normalized to either human *GAPDH* or *HBB* (β-globin) as housekeeping genes. Ct values below detection threshold were ultimately defined as C_t_ = 35.

### Sandwich immuno-sorbent assay (ELISA)

Human CCL2 and human CSF-1 were analysed in cell culture supernatants from A549 control and A549 co-cultures, using the Human CCL2/MCP-1 (R&D Systems, #DCP00) and Human M-CSF/CSF-1 (R&D Systems, #DMC00B) ELISA kits, according to the manufacturer's instructions.

### THP-1 migration assay

Chemotaxis experiments were performed, using a MultiScreen 96-well plate (Millipore, Burlington, MA, USA, #MAMIC5S10), as described before (Gouwy et al. 2008). THP-1 cell migration through the 96-well filter plate occurs in response to a chemotactic gradient. First, the bottom side of the plate was filled with 150 ml of CCL2 (1–30 ng/ml; positive control) diluted in chemotaxis buffer (RPMI without phenol red and L-glutamine, supplemented with 0.1% bovine serum albumin), or with supernatants collected from A549 MT-2 co-cultures at different time points or at varying cell ratios. After placing the 96-well filter plate (5 mm pore size) on top, 100 µl of THP-1 cells at a concentration of 3.5×10^6^ cells per mL were seeded into the upper chamber. After a 3 h incubation at 37 °C, the filter plate was carefully removed and discarded. Migrated THP-1 cells in the bottom plate were quantified using the luminescence ATP detection assay system (Revvity, #6016943). The bottom plate was centrifuged at 1200 × g for 5 min. Then, 50 µl of solution was carefully removed from the bottom plate and replaced with 50 µl of lysis buffer (at RT). The resulting 150 µl mixture (chemokine solution and lysis buffer) was transferred to a “view white” plate (Revvity, Waltham, MA, USA), and the plate was incubated on a shaker for 5 min at 400 x g. After adding 50 µl substrate solution (ATPlite, Revvity, #6016943) and shaking the plate again for 5 min at 400 x g, the plate was incubated in the dark at RT for 10 min, and reading of emitted luminescence was performed using ClarioStar Plus (BMG LabTech).

A chemotaxis index (CI) was calculated by dividing the luminescence value of the test sample by the luminescence value of the control buffer (*n* = 9 in 3 independent biological replicates per condition, padj < 0.05). Obtained CI were normalized to the A549 control condition and represented as log2-transformed values (Mean ± SEM).

### Differentiation of THP-1 cells and primary monocytes

To evaluate the effects of medium-derived chemokines on monocyte differentiation, THP-1 cells were cultured at 2x10^5^ cells per well in 6-well plates, either in RPMI control medium and conditioned medium from HTLV-1-infected (MT-2 SN) or non-infected (Jurkat SN) cells (collected 3 days after passage) (*n* = 3−4). All cultures were performed in a 1:1 ratio (i.e. 1 mL control medium + 1 ml conditioned cell culture SN). Cultures were maintained for 5 days, monitoring cell viability and confluency. Afterwards, THP-1 phenotype was examined microscopically, and macrophage markers were measured by RT-qPCR, following the method described above (Key resources Table). Similar experiments were performed on purified monocytes (*n* = 7), seeded at 1x10^6^ cells per well in 6-well plates, to verify the effects of cell culture-conditioned medium on primary cell differentiation.

To assess the role of the secretomes obtained from A549 co-culture SN, A549 control or A549 NF-kB p65KO cells were co-cultured with MT-2 or Jurkat cells for 48 h in conditions like those described in section 5. After two days, co-culture SN was filtered and used in THP-1 differentiation assay as described above (*n* = 4).

### Statistical analysis

Statistical analyses were performed with Graphpad Prism (v9.5.1), whereas visualization of data was made in R software (v4.4.2). One-way ANOVA or non-parametric Kruskal-Wallis tests were used according to normality testing. Correlation analyses were performed using the Spearman correlation approach. Two-way ANOVA was used to compare A549 genotype (WT vs p65KO) and co-culture conditions (Control, Jurkat SN, MT-2 SN) as independent factors. Post hoc multiple-comparison testing was conducted using Sidak’s correction. For all statistical tests, an adjusted *p* value < 0.05 was the criterion for statistical significance. * = *p* < 0.05; ** = *p* < 0.01; *** = *p* < 0.001; **** = *p* < 0.0001. The tests used for each individual plot are mentioned in the figure legends.

## Results

### Exposure to HTLV-1-infected cells or their supernatant reprograms the transcriptome of A549 alveolar epithelial cells

The lung constitutes one of several organs likely to be affected by HTLV-1-mediated inflammation. To evaluate the impact of HTLV-1 exposure on gene expression in lung epithelial cells, bulk RNA sequencing was performed on A549 cells co-cultured with HTLV-1-infected (MT-2, MT-4) or uninfected (Jurkat) T cells for 24 h (Supplementary Tables 2-6). MT-2 cells have been reported to be high-level producers of HTLV-1, while MT-4 cells do not produce detectable virus (Datta et al. 2000). In parallel, A549 cells were exposed to supernatant (SN) from MT-2 or MT-4 cell cultures to determine the gene expression differences driven by factors like cytokines in the SN of HTLV-1-infected cells.

**Fig. 2:**
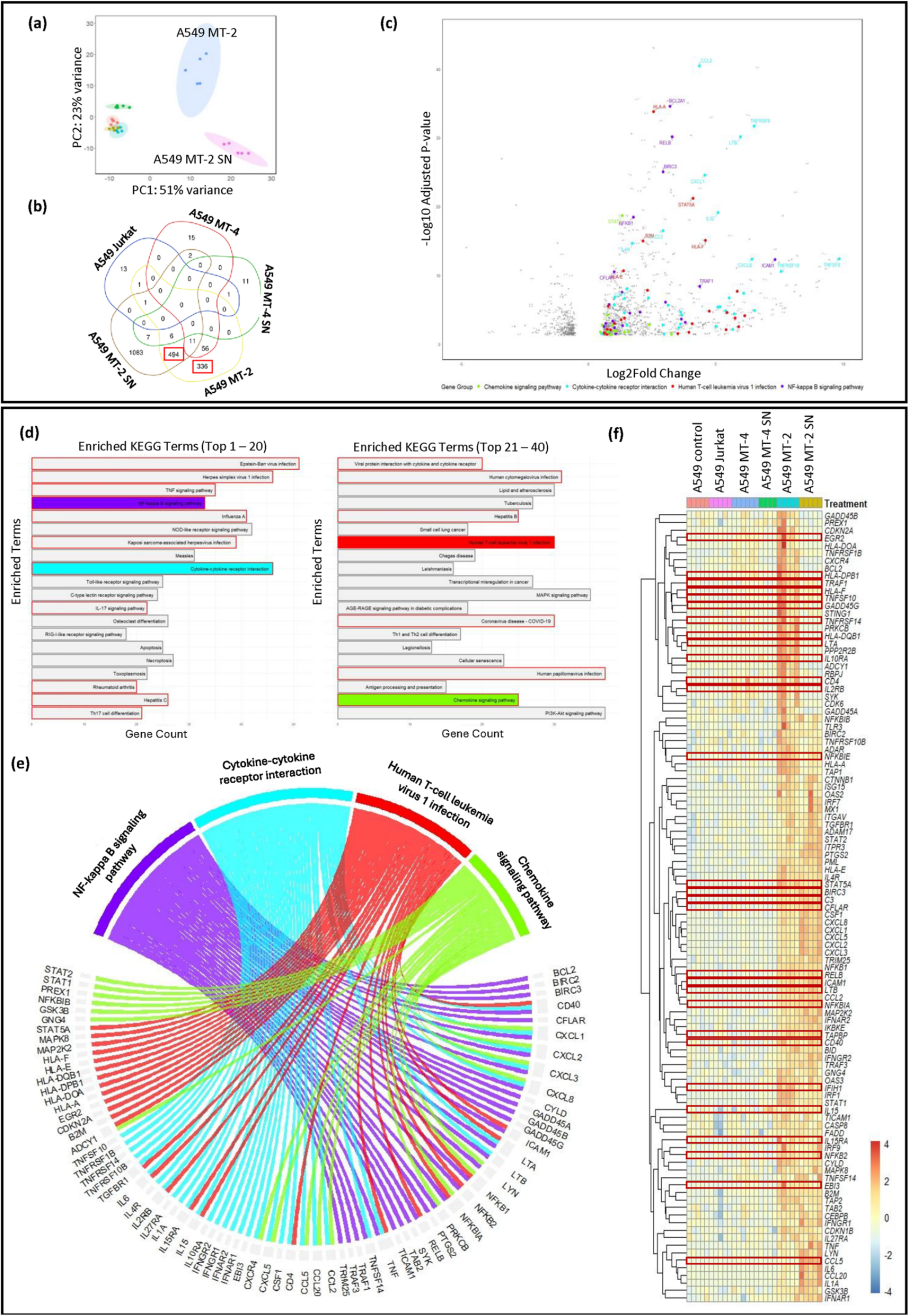
HTLV-1-infected lymphoid cells induce transcriptomic changes in A549 lung epithelial cells. A549 cells (4×10^5^) were co-cultured with Jurkat, MT-4 (or SN), or MT-2 (or SN) cells at a 1:1 ratio for 24 h (*n* = 4–6), and RNA was extracted for sequencing. **a** PCA shows distinct clustering of A549 cells (orange), and A549 cells co-cultured with MT-2 cells (blue), MT-2 SN (purple), MT-4 cells (green), MT-4 SN (turquoise) and Jurkat cells (red). **b** Venn diagram highlights unique and overlapping upregulated DEGs, with MT-2 cells and MT-2 SN showing distinct profiles. **c** Volcano plot displays DEGs in A549 MT-2 vs A549 Jurkat controls. **d** KEGG over-representation analysis of 830 MT-2–specific DEGs (highlighted in red in Figure 2b). Top 40 enriched pathways are shown, with selected pathways highlighted in red. **e** Chord diagram maps 105 clinically relevant DEGs shared across 4 of 18 selected KEGG pathways. **f** Heatmap shows fold changes of the 105 filtered genes across all co-culture conditions. Genes highlighted in red were shown to be positively regulated by HTLV-1 Tax^67^

Principal Component Analysis (PCA) revealed treatment-specific clustering, with clear distinction between the different co-culture conditions (Figure 2a). Sequencing reads were aligned to both the human genome and HTLV-1 reference genome J02029.1. Notably, alignment to the HTLV-1 genome highlighted the presence of viral reads (e.g., reads aligning to HTLV-1 *Gag* and *Pol* sequences) in A549 cells co-cultured with MT-2 cells (Table 2). In contrast, only a small number of HTLV-1-mapped reads were detected in the A549 MT-4 co-cultures, which aligned with RT-qPCR results (Supplementary Figure 1c). Reads mapped to the human genome were subsequently used for differential gene expression analysis (Figure 2, Supplementary Figure 1).

**Table 2:** Alignment of obtained RNA sequencing reads to reference HTLV-1 genome

**HTLV-1 gene**	**mRNA reads**^**a**^				
	**A549 Jurkat**	**A549 MT-4**	**A549 MT-4 SN**	**A549 MT-2**	**A549 MT-2 SN**
Gag	0	0.2	0	2.2	0.6
Pro	0	0	0	0.2	0.2
Pol	0.2	0.7	0.3	22.8	4.2
Rex	0	0	0	0	0
Tax	0	0	0	0	0
Env	0	0	0	0	0
Hbz	0	0	0	0.6	0

^a^Average of mRNA reads obtained from the different A549 co-cultures mapped to an annotated HTLV-1 reference genome (n= 4–6 biological replicates per condition)

DESeq2 software was used to compare gene expression in A549 cells co-cultured with HTLV-1-infected cells, their SN, or non-infected cells, across an average of 14,400 genes detectable above background (Table 3). To confirm that the RNA originated from A549 cells, the DEGs were cross-referenced with known alveolar epithelial markers (Supplementary Figure 2). Cluster analysis of these markers across the different samples showed no noteworthy differences between treatment groups, indicating overall sample homogeneity (Supplementary Figure 2). In line with the PCA analysis, both Venn diagrams and volcano plots confirmed a distinct transcriptional profile observed in A549 cells co-cultured with MT-2 cells, compared to both A549 co-cultures with Jurkat or MT-4 cells (Figures 2b-c, Supplementary Figures 1a-1b).

**Table 3:** Differential gene expression analysis

**Condition**	**DEGs**^**a**^	**Upregulated DEGs**^**b**^	**Downregulated DEGs**^**b**^
A549 Jurkat vs A549 control	0.7% (103/13742)	16% (16/103)	85% (87/103)
A549 MT-2 vs A549 Jurkat	8.5% (1304/15286)	69% (905/1304)	31% (399/1304)
A549 MT-2 SN vs A549 Jurkat	20% (2956/14900)	54% (1604/2956)	46% (1352/2956)
A549 MT-4 vs A549 Jurkat	0.6% (90/15673)	93% (84/90)	6.6% (6/90)
A549 MT-4 SN vs A549 Jurkat	0.6% (80/12586)	31% (25/80)	69% (55/80)

^a^Percentage of significantly DEGs in the different A549 co-cultures (padj <0.5). Data was normalized to the total number of transcripts, and A549 Jurkat co-culture was used as reference (except for A549 Jurkat, where A549 monoculture was used as reference)

^b^Percentage of significantly upregulated or downregulated genes within the statistically deregulated genes measured in the different A549 co-cultures

HTLV-1 spreads primarily via cell-to-cell contact. While co-culture systems accurately model this process, it often results in complex mixtures of donor and target cell materials, complicating downstream analyses. Residual HTLV-1-infected cells may adhere to A549 cells, obscuring epithelial-specific transcriptomic changes. To address this, transcriptome deconvolution was performed using CIBERSORTx (Newman et al. 2019) to quantify potential contamination by MT-2 or MT-4 transcripts, identified through digital transcriptomics (Vanderlinden *et al.*, unpublished data) (Supplementary Figure 1d). CIBERSORTx is a computational algorithm that estimates the relative proportions of different cell types in bulk RNA-seq data. By deconvolving gene expression profiles using reference cell-type signature matrices, this approach allows for the detection and quantification of distinct cellular contributions within complex tissue samples. Thus, this allows for accurate quantification of possible contribution of remaining MT-2- or MT-4-specific RNAs among the DEGs in the A549 co-culture or SN conditions. As shown in Supplementary Figure 1d, no significant increase in MT-2 or MT-4-specific transcripts were observed across all experimental conditions.

While only 80–103 (0.6–0.7.6.7%) DEGs were identified (padj <0.05 after stringent FDR correction) in Jurkat and MT-4 conditions, 1304 (8.5%) and 2956 (19.8%) genes were significant in A549 MT-2 and A549 MT-2 SN co-cultures, respectively (Table 3). Most DEGs in A549 MT-2 or MT-2 SN conditions were unique, with 336 (37%) and 1083 (68%) of upregulated genes exclusive to each treatment, respectively (Figure 2b). In contrast, A549 co-cultured with MT-4 cells or MT-4 SN displayed similar transcriptomic profiles to A549 Jurkat control (Figures 2a-2b, Supplementary Figures 1a-1b, Table 3). Interestingly, SN exposure induced stronger gene downregulation, with 46% of downregulated DEGs in A549 cells exposed to MT-2 SN compared to 31% in MT-2 co-culture settings (Table 3). Log2 fold changes and adjusted *p*-values of the 20 most significant DEGs (padj < 0.05) per condition are summarized in Supplementary Table 9. For downstream analysis, we focused on the 830 genes significantly upregulated in A549 cells co-cultured with MT-2 cells (Figure 2b, red boxes), enabling the characterization of key transcriptomic changes induced by HTLV-1 exposure.

A systems biology analysis was performed on these 830 selected DEGs to identify key biological pathways influenced by exposure of the A549 cells to HTLV-1 (Supplementary Table 10). The 830 DEGs were mainly linked to various viral infections, as shown by the significant enrichment of KEGG terms, such as “Human T-cell leukemia virus 1 infection,” “Epstein-Barr virus infection,” and “Hepatitis B/C infection” (Figure 2d), all linked to cancer and/or (neuro) inflammation. Complementary Gene Ontology (GO) enrichment analysis confirmed enrichment in terms such as "Defense Response to Virus" and "Viral Process" (Supplementary Figure 3). Moreover, KEGG over-representation analysis revealed strong activation of inflammatory pathways, including “TNF signaling pathway”, “NF-kappa B signaling pathway” and “IL-17 signaling pathway” (Figure 2d). These observations were consistent with elevated pro-inflammatory cytokine levels measured in A549 MT-2 co-cultures (Supplementary Figure 5a-5d). In addition to inducing inflammation, HTLV-1 exposure also activated both innate and adaptive immune responses in A549 cells, as revealed by enrichment in pathways, such as “Toll-like receptor signaling” and “Cytokine-cytokine receptor interactions” (Figure 2d). Of note, the upregulation of the “Chemokine signaling pathway” suggested the enhanced interplay between HTLV-1-exposed A549 cells and nearby immune cells during HTLV-1 infection (Figure 2d). This finding was supported by GO analysis, which revealed enrichment of pathways related to "Leukocyte chemotaxis", "Monocyte differentiation" or “Macrophage activation”, indicating immune cell engagement at sites of HTLV-1 exposure (Supplementary Figure 4, Supplementary Table 11).

**Fig. 3:**
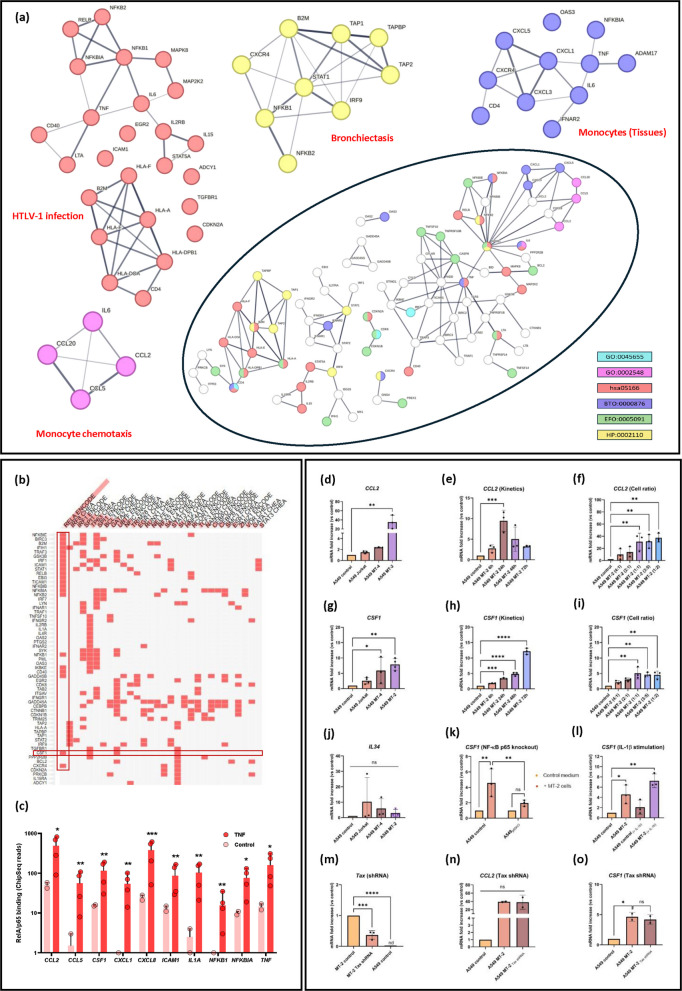
HTLV-1 infection promotes pro-inflammatory signaling and immune activation in lung epithelial cells. Among 830 upregulated genes in A549 MT-2 cells, 105 genes were selected based on overlap with at least 2 of 18 enriched KEGG pathways. **a** PPI network analysis grouped the associated proteins into 5 biological processes, highlighting enrichment in HTLV-1 infection (hsa05166, red), monocyte differentiation (GO:0045655, cyan), monocyte recruitment (GO:0002548, magenta), tissue-resident monocyte activity (BTO:0000876, dark blue), and bronchiectasis-related inflammation (HP:0002110, yellow). **b** Transcription factors (TFs) regulating the 105 selected genes were identified through Upstream Transcription Factor enrichment analysis. **c** RelA (NF-κB p65) chromatin binding dynamics at key NF-κB gene loci in BEAS-2B airway epithelial cells. Publicly available ChIP-seq data (GSE79803) were reanalyzed to compare RelA occupancy under control condition or after TNF stimulation (20 ng/mL) for 1 h. **d**–**i** RT-qPCR showed cell ratio- and time-dependent increases in *CCL2* and *CSF-1* expression following MT-2 co-culture (*n* = 3). **j**-**l** In A549 p65KO cells, *CSF1* upregulation was suppressed, implicating canonical NF-κB signaling (*n* = 3). **m**–**o ***Tax* knockdown in MT-2 cells did not significantly alter *CCL2* or *CSF1* expression in A549 cells (*n* = 2). Statistical significance: one-way ANOVA with Tukey post hoc test; *p < 0.05, **p < 0.01, ***p < 0.001, ****p < 0.0001

To dive deeper into the biological processes and disease-associated pathways, the 830 upregulated DEGs from A549 MT-2 co-cultures (Figure 2b) were filtered based on 18 KEGG pathways clinically relevant to HTLV-1 infection (i.e., pathways associated with oncogenic, (neuro)inflammatory, or respiratory viral infections) (Figure 2d, Supplementary Table 10). This biological filtering process yielded 105 of the 830 upregulated DEGs (Figure 2f). To illustrate their distribution across a subset of selected pathways, a Circos plot was generated, providing a global overview of the pathway-gene relationships (Figure 2e). These genes were subsequently used to construct a PPI network, which highlighted hub proteins essential for crucial cellular processes and bottleneck proteins known to regulate multiple pathways simultaneously (Figure 3a). Notably, 25 of these proteins were significantly enriched in the KEGG pathway “Human T-cell leukemia virus type 1 infection”, supporting the relevance of the experimental model. STRING analysis further highlighted enrichment in terms such as “Tissue monocytes” and “Bronchiectasis”, aligning with KEGG results (Supplementary Table 10), previously reported clinical data, and recent multi-omics findings (Figure 3a) (Einsiedel et al. 2012; Honarbakhsh and Taylor 2015; Van Weyenbergh et al. Van 2025).

Although CD4^+^ T cells are the primary target of HTLV-1 infection, monocytes are also potential candidates. Epithelial pro-inflammatory signaling enhances cytokine and chemokine production, thereby promoting the recruitment of immune cells such as monocytes to the lung. Monocytes migrate to inflamed tissues in response to these signals and differentiate into macrophages, whose functional phenotype is shaped by the local microenvironment and can contribute either to antiviral defense or tissue repair (Watanabe et al. 2019; Dias et al. 2021; Wynn et al. 2013).

The role of monocyte recruitment in virus-induced pulmonary inflammation was further supported by the enrichment of the GO term “Monocyte chemotaxis” in the PPI network, driven by key chemokines such as CCL2, CCL5, CCL20, and the cytokine IL-6 (Figure 3a). Thus, upon recruitment to the lungs, monocytes might undergo differentiation into inflammatory or profibrotic macrophages, as indicated by enrichment of the term “Regulation of monocyte differentiation” and increased expression of growth factors, like CSF-1 (Figure 2f). Activation of the CSF-1R signaling axis was further supported by enhanced presence of JAK/STAT pathway components, including STAT1, STAT2, and STAT5A. Notably, STAT5A is known to promote expression of the anti-apoptotic gene *BCL2*, which was also present among the filtered KEGG gene list, suggesting a potential mechanism for increased cell survival during infection (Figures 2f, 3a).

### Exposure to HTLV-1 drives the development of a pro-inflammatory alveolar microenvironment and leads to recruitment of immune cells to the lungs

Given the association of HTLV-1 exposure with pulmonary inflammation, immune cell infiltration and the activation of NF-κB signaling in HTLV-1-exposed epithelial cells (Figure 2c), we next investigated whether HTLV-1 induces a pro-inflammatory response in A549 cells. To address this, A549 cells were co-cultured for 48 h with HTLV-1-infected MT-2 or MT-4 cells, or with uninfected Jurkat cells, and mRNA levels of key pro-inflammatory cytokines (e.g., *IL6*, *CXCL8*, *IL1B*, *TNF*) were measured (Supplementary Figures 5a–5d). For all these cytokines, a significant increase in expression was observed in A549 cells co-cultured with MT-2 cells, consistent with RNA-seq data (Figure 2f). In parallel, mRNA levels of important chemokines and growth factors involved in immune cell recruitment and myeloid cell maintenance, including *CCL2*, *CSF1*, and *IL34*, were assessed in HTLV-1-exposed epithelial cells (Figures 3d, g, j).

Among other HTLV-1-induced factors, CSF-1, a key regulator of monocyte proliferation and differentiation, was significantly upregulated in A549 cells co-cultured with MT-2 cells (Figure 3g). In contrast, mRNA levels of IL-34, another ligand of the CSF-1R, remained unchanged across all conditions, confirming the selective induction of *CSF1* following HTLV-1 exposure (Figure 3j). Interestingly, *CSF1* expression was maximal at an A549:MT-2 cell ratio of 1:1 (Figure 3i), and increased over time, reaching a 13-fold rise at 72 h (Figure 3h).

CCL2 is a chemokine that plays a key role in the immune response by acting as a chemoattractant, primarily recruiting monocytes and other immune cells to sites of inflammation or tissue injury. In line with RNA-seq data (Figure 2f, Supplementary Table 9), A549 cells co-cultured with MT-2 cells showed a significant increase in *CCL2* levels compared with A549 cells alone (Figure 3d). *CCL2* expression was maximal at 24 h incubation, and increased with higher MT-2 cell number, indicating a cell ratio- and time-dependent regulation (Figures 3e -3f). Together, these findings indicate that HTLV-1 exposure promotes the expression of both CCL2 and *CSF-1*, key mediators of monocyte recruitment to the lung epithelium (Figures 3d-i).

Upstream transcription factor (TF) enrichment analysis, using the ENCODE database, was performed on the 105 KEGG-filtered genes to identify principal transcriptional regulators. This systems-level approach revealed several NF-κB-related TFs, with RelA (NF-κB p65) emerging as a prominent regulator of the selected genes (Figure 3b). This TF regulates the expression of important pro-inflammatory cytokines like CCL2, as well as important growth factors such as CSF-1. Complementary analysis using the ARCHS4 Tissue database further indicated that our KEGG filtered gene list (Figure 2f) was predominantly regulated by TFs active in macrophages, including alveolar macrophages (Supplementary Figure 5e).

To validate and extend these findings in a lung epithelial pro-inflammatory context, publicly available NF-κB RelA ChIP-seq datasets from BEAS-2B airway epithelial cells were reanalyzed to characterize RelA chromatin binding dynamics under inflammatory stimuli (Figure 3c) (Kadiyala et al. 2016). Under baseline (control) conditions, RelA binding was generally low across all examined loci, consistent with minimal NF-κB activity. In contrast, following 1 h of TNF treatment, a marked increase in RelA occupancy was observed at promoters and regulatory regions of canonical NF-κB target genes, such as *NFKB1* and *NFKBIA*, as well as at loci encoding pro-inflammatory chemokines (e.g., *CCL2*, *CCL5*) and cytokines/growth factors (e.g., *IL1A*, *CSF1*). This enhanced RelA binding activity confirmed a robust NF-κB activation in alveolar epithelial cells under pro-inflammatory conditions (Figure 3c).

To confirm whether *CSF1* upregulation is, at least partially, driven by NF-κB activation (Figures 3b-c), an A549 NF-κB RelA/p65 knockout cell line (A549 p65KO) was generated using CRISPR-Cas9. RelA knockout was validated by Western blot and indirectly by flow cytometric assessment of ICAM-1 expression in A549 cells (Supplementary Figures 5f-5g). Upon co-culture with MT-2 cells, A549 p65KO cells did not exhibit significant *CSF1* induction (Figure 3k). Instead, a significant decrease in *CSF1* expression was measured when compared to A549 control MT-2 condition. In addition, IL-1β stimulation enhanced *CSF1* expression in A549 control cells (Figure 3l), supporting the pro-inflammatory role of NF-κB activation in lung epithelial cells. These results are consistent with the RelA ChIP-seq data (Figure 3c), which demonstrated increased RelA binding at the *CSF1* promoter region under pro-inflammatory conditions. Finally, the contribution of the HTLV-1 transactivator Tax protein in regulating *CSF1* and *CCL2* expression was assessed to determine whether viral protein expression could directly impact their expression levels. However, both *CSF1* and *CCL2* expression remained unchanged in A549 cells co-cultured with MT-2 cells expressing *Tax-*targeting shRNA (Figures 2f, 3n-3o). This finding supports prior transcriptomic results from Jurkat cells over-expressing *Tax*, which showed that *Tax* does not regulate *CSF1* expression (Figure 2f) (Vandermeulen et al. 2021).

**Fig. 4:**
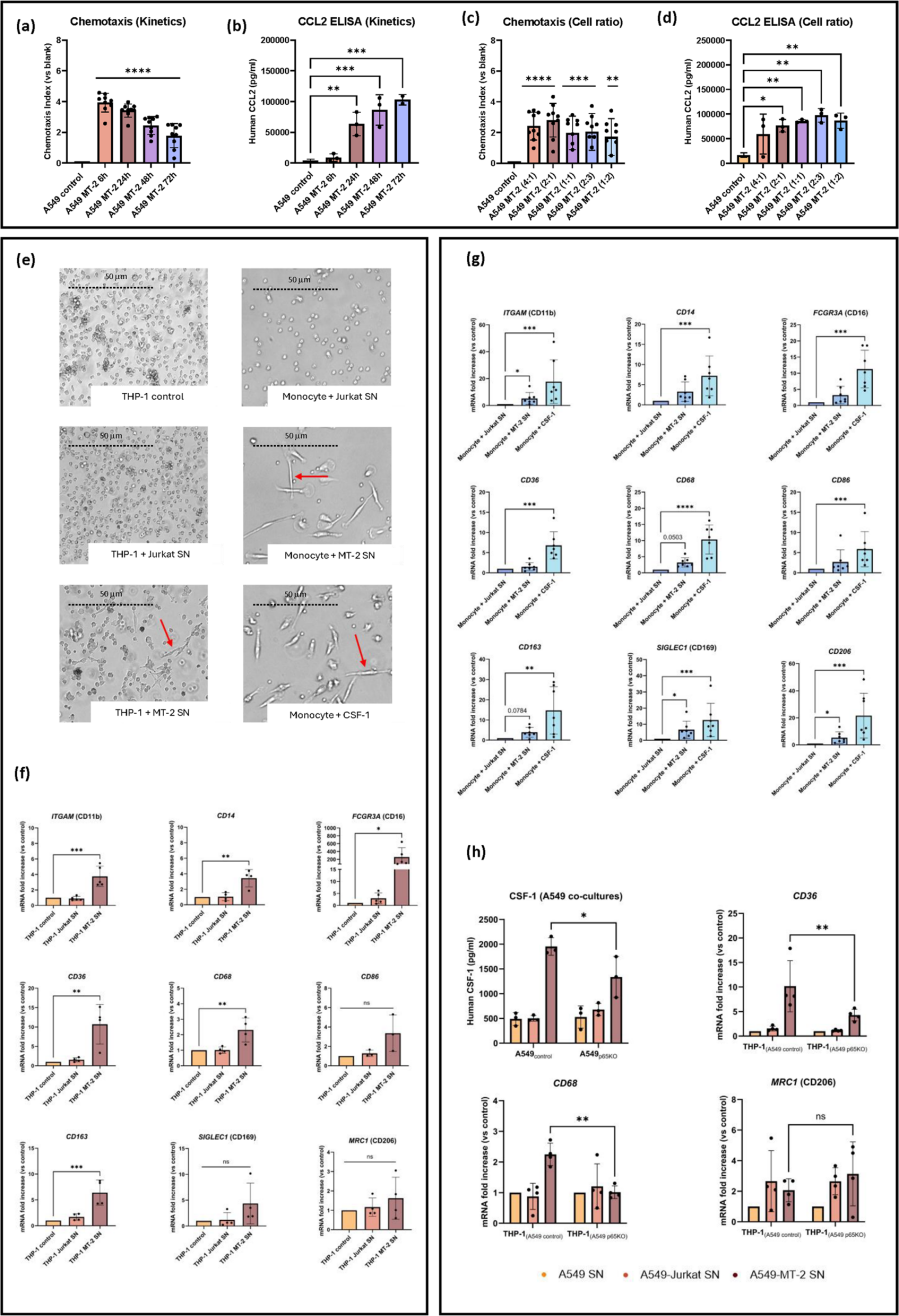
HTLV-1-induced factors drive monocyte recruitment and macrophage differentiation. **a**–**d** A549 cells were co-cultured with MT-2 cells at different ratios and for varying durations. **a**, **c** SN from these cultures was used in THP-1 chemotaxis assays **b**, **d**, and CCL2 levels were quantified by ELISA. Peak THP-1 migration was observed for SN harvested at 6 h, despite lower CCL2 levels. **e** SN from MT-2 cell cultures induced elongation and flattening in THP-1 cells and primary monocytes, resembling CSF-1-treated monocytes. **f** THP-1 cells cultured in MT-2 SN for 5 days showed increased expression of macrophage markers, as quantified by RT-qPCR (*n* = 3–4), supporting monocyte differentiation. **g** CD14^+^ monocytes (*n* = 7) were cultured with Jurkat SN, MT-2 SN, or CSF-1 (50 ng/mL) . RT-qPCR confirmed macrophage marker induction. **h** CSF-1 levels in the SN of A549 control or A549 p65KO cells co-cultured with Jurkat or MT-2 cells were quantified by ELISA (*n* = 3). THP-1 cells incubated with the SN of A549 MT-2 co-cultures for 5 days showed upregulation of macrophage markers, indicative of monocyte differentiation (*n* = 4). This upregulation was significantly decreased in THP-1 cells cultured with SN from A549 p65KO MT-2 co-cultures. Statistical significance: one-way ANOVA with Tukey post hoc test for THP-1 RT-qPCR; Kruskal-Wallis used for primary monocytes RT-qPCR.; Two-way ANOVA with genotype and co-culture condition as factors, followed by Sidak’s multiple-comparison test for CSF-1 ELISA and THP-1 differentiation assays with co-culture SN. *p* < 0.05.; *p < 0.05, **p < 0.01, ***p < 0.001

To evaluate the role of HTLV-1 in monocyte recruitment, chemotaxis assays were performed using THP-1 monocytic cells exposed to increasing concentrations of CCL2 (1–30 ng/mL). CCL2 clearly induced cell migration at concentrations ≥10 ng/mL (Table 4). Consistently, stimulation with SN from uninfected Jurkat cells failed to promote THP-1 migration (chemotactic index [CI]: 0.8 ± 0.8, n = 3), whereas SN from HTLV-1-infected MT-2 cells induced robust chemotaxis (CI: 3.6 ± 1.3, n = 3) (Table 4). In parallel, assays using SN from A549 MT-2 co-cultures revealed a time-dependent increase in cell migration (Figure 4a), which correlated with increased CCL2 levels in the SN (Figure 4b). Interestingly, THP-1 migration was highest for SN harvested at 6 h (CI: 3.9 ± 0.1, n=9) and declined substantially by 48 h (CI: 2.4 ± 0.2, n = 9) (Figure 4a, Table 4), indicating that migration peaked early on at suboptimal concentrations of CCL2 (±10 ng/mL) (Table 4). Similarly, increasing the number of MT-2 cells in co-culture led to higher CCL2 concentrations in the SN (Figure 4d), while chemotaxis peaked at an A549:MT-2 ratio of 2:1 (Figure 4c). As CCL2 levels continued to rise, THP-1 chemotactic responsiveness declined (Figures 4c, d, Table 4), suggesting that excessive chemokine concentrations may desensitize monocytes to chemotactic gradients.

**Table 4:** Supernatant of A549-MT-2 co-culture induces chemotaxis of THP-1 cells

**Chemokine/Condition**	**Concentration (ng/ml)**^**a**^	**Chemotaxis Index**^**b**^
CCL2	1.0	1.4 ± 0.3 ns
	3.0	2.4 ± 1.4 ns
	30	4.1 ± 2.5 *
RPMI control	NA	0
Jurkat SN	NA	0.8 ± 0.8 ns
MT-2 SN	NA	3.6 ± 1.3 **
A549 control (6h)	3.8	0
A549 MT-2 (6h)	9.7	3.9 ± 0.1 ****
A549 MT-2 (24h)	64	3.4 ± 0.1 ****
A549 MT-2 (48h)	85	2.4 ± 0.2 ****
A549 MT-2 (72h)	73	1.8 ± 0.2 ****
A549 control (Cell ratio)	18	0
A549 MT-2 (4:1)	60	2.4 ± 0.2 ****
A549 MT-2 (2:1)	76	2.8 ± 0.3 ****
A549 MT-2 (1:1)	85	2.0 ± 0.6 ***
A549 MT-2 (3:5)	97	2.0 ± 0.6 ***
A549 MT-2 (1:2)	86	4.2 ± 0.6 **

^a^CCL2 concentration present in the different cell culture supernatants was measured by ELISA (*n* = 3 biological replicates per condition)

^b^Chemotaxis Index was calculated by dividing the luminescence value of the test sample by the luminescence value of the control buffer. Obtained CI were normalized to the A549 control condition and represented as on graphs as log2-transformed values (Mean ± SEM). (*n* = 9 in 3 independent biological replicates per condition, padj < 0.05). Statistical significance: one-way ANOVA with Tukey post hoc test; *p < 0.05, **p < 0.01, ***p < 0.001, ****p < 0.0001

To explore the effects of soluble factors secreted by HTLV-1-infected cells on monocyte recruitment and differentiation, THP-1 monocytic cells were cultured for five days in either standard medium or medium conditioned by Jurkat or MT-2 cell cultures (Figures 4e-f). THP-1 cells exhibited notable morphological changes when cultured with MT-2 SN, adopting an elongated shape, and becoming adherent (Figure 4e). To further characterize these polarized cells, RNA was extracted from various THP-1 co-cultures, and RT-qPCR was performed to assess the levels of different macrophage markers expressed on their surface (Figure 4f). THP-1 cells exposed to MT-2 SN showed increased mRNA levels of *ITGAM* (CD11b), *CD14*, and *FCGR3A (*CD16*)*, 3 markers commonly associated with myeloid and monocyte lineage. Similarly, elevated expressions of macrophage-specific markers *CD36*, *CD68* and *CD163* were measured, indicating a shift toward a macrophage-like phenotype following HTLV-1 exposure (Figure 4f).

To confirm the clinical relevance of these *in vitro* findings, we validated the expression of key markers identified in THP-1 cells (Figure 4f), using primary monocytes isolated from PBMCs of healthy donors (Figure 4g). Monocytes were purified by negative selection and confirmed by multicolor flow cytometry using CD3 (APC-Cy7) and CD14 (BV421) stainings (Supplementary Figure 5h). The cells were then cultured in conditioned media or stimulated with 50 ng/mL CSF-1 to induce macrophage differentiation. Similar to THP-1 results, primary monocytes exposed to MT-2 SN or CSF-1 underwent notable morphological changes (Figure 4e). Both treatments increased *ITGAM* (CD11b) expression (Figure 4g). While CSF-1 stimulation significantly upregulated all tested macrophage markers (*CD36*, *CD68*, *CD86*, *CD163*, *SIGLEC1* [CD169], and *MRC1* [CD206]), MT-2 SN specifically induced significant increases in *SIGLEC1* (CD169) and *MRC1* (CD206) only (Figure 4g).

Finally, we investigated whether A549 NF-κB activity modulates monocyte differentiation through regulation of CSF-1 protein levels in the supernatant of A549 MT-2 co-cultures (Figure 4h). ELISA analysis demonstrated higher CSF-1 levels in SN derived from A549 control MT-2 co-cultures (1953 ± 102 pg/ml) compared with SN from A549 p65KO MT-2 co-cultures (1337 ± 239 pg/ml), corresponding to a 32% reduction. Two-way ANOVA demonstrated a significant interaction between A549 genotype (control vs. p65KO) and co-culture condition (control, Jurkat, MT-2), indicating that the impact of epithelial NF-κB signaling on CSF-1 production is genotype-dependent and most pronounced under MT-2 stimulation. Next, supernatants from A549 control and A549 NF-κB p65KO cells co-cultured with MT-2 or Jurkat cells were filtered and added to THP-1 cells (Figure 4h). Notably, the reduced CSF-1 abundance in p65KO-derived SN was associated with altered differentiation profiles of THP-1 monocytes, as measured by *CD36* and *CD68* gene expression (Figure 4h). Indeed, we found that the marked upregulation of *CD36* and *CD68* THP-1 differentiation markers (Figure 4f) was significantly blunted by CRISPR/Cas9-mediated RelA/p65 inactivation, confirming our hypothesis.

### Transcriptomic analysis of alveolar epithelial cells identifies multi-omics markers of HTLV-1-associated disease and idiopathic pulmonary fibrosis

**Fig. 5:**
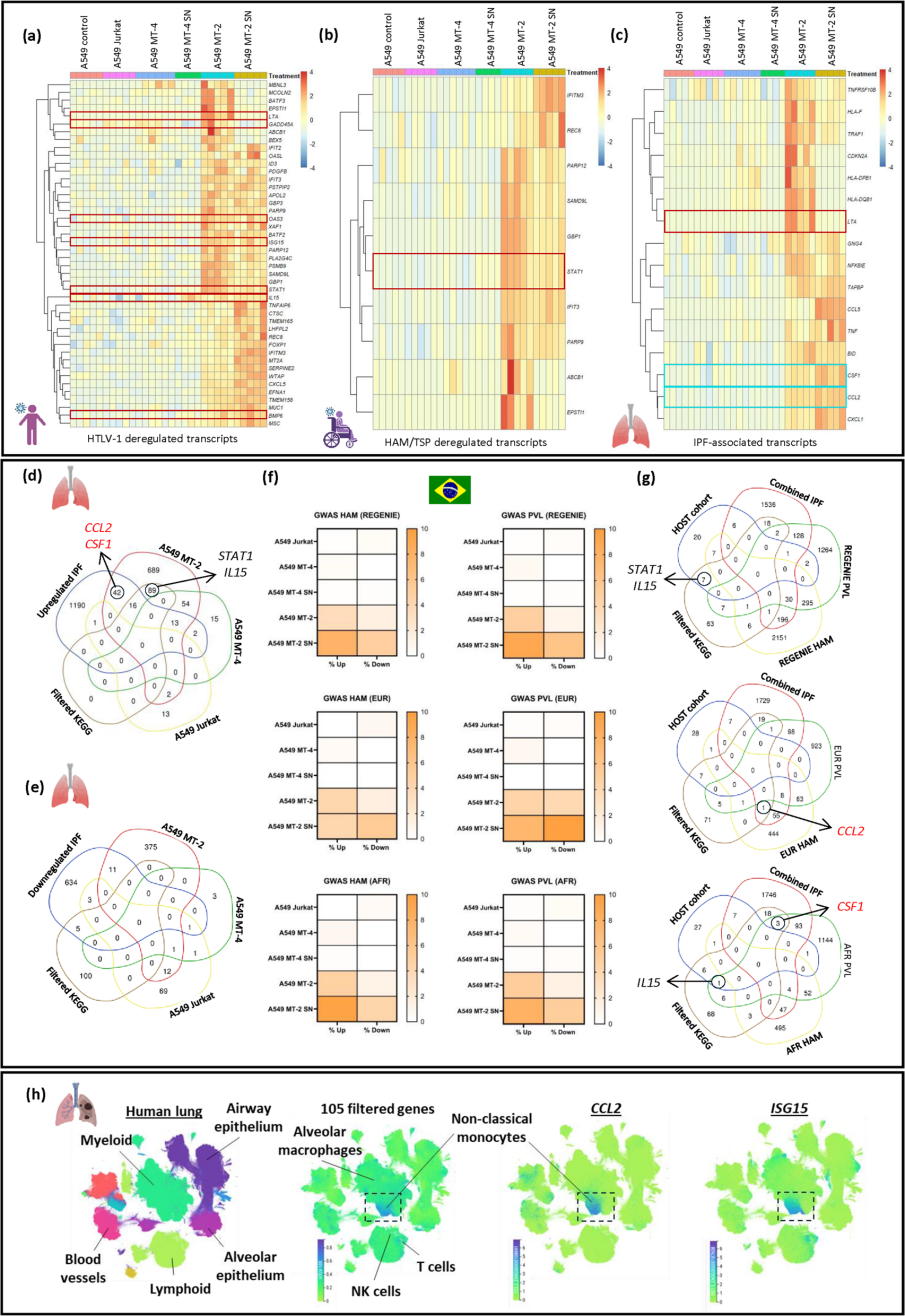
Transcriptomic and genomic overlap between HTLV-1-induced lung inflammation, HAM/TSP, and IFP signatures. **a–b** DEGs (n = 830) from A549 MT-2 co-cultures were compared to HTLV-1-deregulated PBMC transcripts reported by Tattermusch et al (2012), including 80 HAM/TSP-specific genes. Fold changes are shown for overlapping transcripts. Genes highlighted in red are the genes that are also represented in the filtered KEGG gene list. **c** A curated list of IPF-associated genes was compiled from five GEO datsets (GSE32537, GSE47460, GSE53845, GSE70866, GSE110147). Genes significantly deregulated in at least three datasets were cross-referenced with the 105 KEGG-filtered DEGs from A549 MT-2 co-culture. The heatmap highlights shared genes, with those in red representing overlap with the identified HTLV-1-deregulated PBMC transcripts (see Figure 5a), and those in cyan denoting the genes selected as focal points for the *in vitro *experiments (see Figure 3). **d-e **Venn diagrams show overlap between curated IPF gene sets and DEGs from various A549 co-cultures. **f** Heatmaps illustrate overlap percentages between GWAS datasets (multi-ancestry: Regenie software, African and European ancestry: Tractor Software Van Weyenbergh et al 2025) and A549-derived DEGs. **g** Venn diagrams summarize intersections across HOST cohort, IPF-up/downregulated genes, KEGG-filtered genes, and GWAS hits. **h **UMAPs from single-cell data show expression profiles of overlapping genes across key cell types

In the context of HAM/TSP, genome-wide transcriptome analysis of whole blood samples has enabled the identification of disease-specific biomarkers, supporting the development of targeted diagnostics (Tattermusch et al. 2012; Manolio 2010). In this study, DEGs measured from the different A549 co-culture conditions (Supplementary Tables 2–6) were compared to previously published datasets (Tattermusch et al. 2012; Van Weyenbergh et al. 2025; Kwaan et al. 2006; Assone et al. 2022) using systems biology analysis to evaluate their concordance with known HTLV-1-associated biomarkers (Figures 5a- 5b). Specifically, significantly upregulated DEGs in A549 MT-2 co-cultures (Figure 2b, red box) were compared with whole blood transcriptome signatures from Tattermusch *et al. (*Tattermusch et al. 2012) (GSE29312), who reported 542 HTLV-1-deregulated transcripts, including 80 specifically linked to HAM/TSP. Comparative analysis revealed 44 overlapping genes with the HTLV-1 signature profile and 10 with the HAM/TSP-specific subset (Figure 5a). Notably, 7 of the 105 genes from our KEGG pathway enrichment list were found among the 44 shared genes, including *GADD45A*, *LTA*, interferon-regulated genes *OAS3* and *ISG15*, and immune regulators involved in monocyte recruitment and differentiation *STAT1*, *IL15*, and *CXCL5* (Figure 5a). Of interest, *STAT1* was identified as a key HAM/TSP biomarker both *in silico* and *in vivo *(Menezes 2018; Menezes et al. 2017), highlighting its potential role in disease pathogenesis (Figure 5b).

Bronchiectasis is a common clinical manifestation of HAPD and is frequently associated with HTLV-1 infection, particularly in patients with HAM/TSP (Steinfort et al. 2008; Einsiedel et al. 2012; Honarbakhsh and Taylor 2015). This condition is driven by chronic lung inflammation and fibrotic remodeling, processes in which monocyte-derived alveolar macrophages play a key role and are sustained by CSF-1/CSF-1R signaling (Joshi 2020). To investigate fibrotic-like changes induced by HTLV-1 exposure, idiopathic pulmonary fibrosis (IPF) was used as a representative disease model of fibrosis to compare HTLV-1-associated transcriptomic and genomic datasets. A cross-analysis of publicly available transcriptomic datasets was performed, incorporating samples from confirmed IPF patients and healthy donors (GSE32537 (Yang et al. 2013), GSE47460 (Peng et al. 2016; Anathy et al. 2018; Kim et al. 2015; Yu et al. 2018; Tan et al. 2016), GSE53845 (DePianto et al. 2015), GSE70866 (Prasse et al. 2019), GSE110147 (Cecchini et al. 2018)) (Figures 5c-5e). An IPF gene list was compiled through consensus analysis across these datasets, retaining genes that were consistently deregulated in IPF samples in at least three datasets. Genes that appeared in both up- and downregulated sets across datasets were excluded from the final list to ensure robustness. Among our 105 filtered KEGG genes (Figure 2f), 16 overlapped with the IPF-upregulated gene set (Figure 5c), including immune-related chemokines and growth factors such as *CCL2*, *CXCL1*, *CCL5*, and *CSF1*. *TNF*, a key inflammatory regulator, was also commonly upregulated, suggesting a mechanistic link between HTLV-1-induced immune modulation and fibrotic remodeling in pulmonary tissue (Figure 5c).

Beyond comparisons with general HTLV-1 and HAM/TSP transcriptomic signatures (Figures 5a-5b), the same DEGs were cross-referenced with a recent multi-ancestry GWAS (Van Weyenbergh et al. 2025) for both HAM/TSP and proviral load (PVL) (Figure 5f). Incorporation of multi-ancestry GWAS data provides complementary population-level support for our molecular findings and enables ancestry-specific associations of transcriptomic changes. A549 cells co-cultured with MT-2 or exposed to MT-2 SN exhibited transcriptomic profiles that closely aligned with genes identified in multiple GWAS analyses (Figure 5f). Notably, 4–10% of deregulated genes under both conditions overlapped with GWAS findings, highlighting a strong association with both HAM/TSP diagnosis and elevated HTLV-1 PVL (Figure 5f, Supplementary Figure 7). Key overlapping genes included regulators critical for monocyte recruitment and macrophage differentiation, such as *CCL2* in the European cohort and *CSF1* in the African cohort (Figure 5g).

Genes identified in multi-ancestry GWAS (Figure 5f) were compared to the IPF gene list, focusing on significantly upregulated genes (Figure 5g). These genes were cross-referenced with our filtered KEGG gene list (Figure 2f) and an additional *ex vivo* trancriptomic dataset (HOST cohort) (Kwaan et al. 2006; Assone et al. 2022) obtained using a different platform (nCounter, Nanostring, Supplementary Figure 6). The HOST cohort provides unique transcriptional data supported by longitudinal follow-up of people living with HTLV-1. Notably, this cohort reported incident HAM/TSP cases identified through routine blood donor screening, enabling the study of transcriptomic changes at disease onset and the evaluation of disease progression dynamics over time. Venn diagram analyses revealed significant overlap among the filtered KEGG gene list, the IPF gene set, and the *ex vivo* HOST cohort (Figure 5g). Notably key immune-related regulators, including *STAT1* or *IL15*, were found in both the filtered KEGG gene list and the *ex vivo* HOST dataset. These genes play a central role in interferon signaling, monocyte activation, and antiviral defense (Figure 5g). Consistently, these results supported the protein-protein interaction (PPI) network shown in Figure 3a, where several of these regulators appeared as central nodes in pathways enriched across both KEGG and GWAS analyses (e.g., *STAT1*, *TNF*). Together, this convergence of transcriptomic and genomic evidence highlights the potential involvement of these regulators in HAM/TSP pathogenesis and progression.

The 105 filtered KEGG genes were further analyzed using single-cell RNA-seq data from the integrated Human Lung Cell Atlas (HLCA) (Sikkema et al. 2023) (Figure 5h). The HLCA is an open-access resource comprising over 2 million respiratory tract cells collected from 486 individuals, encompassing 49 distinct datasets. Its core includes data from healthy lung tissue, which can be directly compared to samples from individuals with various lung diseases. Analysis showed that expression of the 105-gene signature (Figure 2f) was elevated in multiple inflammatory lung conditions, such as pulmonary fibrosis, chronic obstructive pulmonary disease, and COVID-19 (Supplementary Figure 8). Additionally, cross-comparison identified a distinct myeloid cell subset characterized by high *CCL2* expression, which aligned with the known HAM/TSP type I interferon (IFN) gene signature (Figure 5h, Supplementary Figure 8). Notably, this specific myeloid subset was characterized by a strong correlation between *CCL2* and *ISG15/CXCL10* expression (Supplementary Figures 8b-8c), two IFN-regulated genes commonly upregulated in HAM/TSP patients, of which CXCL10 has been validated as a bona fide biomarker for clinical evolution in HAM/TSP (Da Silva et al. 2025; Tamaki et al. 2019; Yamauchi et al. 2021; Yamauchi et al. 2020).

## Discussion

While HTLV-1 tropism for lung tissues is well established (Einsiedel et al. 2021; Kimura et al. 1986; Sugimoto et al. 1987; Normando et al. 2020), its interaction with non-lymphoid cells, particularly epithelial cells, remains unclear. Previous *in vitro* studies demonstrated that alveolar epithelial cells could in fact harbor HTLV-1, as shown by the detection of proviral DNA and viral proteins in A549 cells, following exposure to HTLV-1-infected cells (Teruya et al. 2008). In this study, we assessed the effects of HTLV-1 exposure on A549 cells, using a multi-omics approach. To investigate the cellular mechanisms underlying the chronic inflammation characteristic of HAPD, transcriptomic profiling was performed on A549 cells following co-cultures with HTLV-1-infected MT-2 or MT-4 cells, or non-infected Jurkat cells (Supplementary Tables 2–6). In parallel, A549 cells were also stimulated with MT-2 or MT-4 SN to determine the impact of HTLV-1-associated soluble factors on gene expression. DEGs analysis revealed stimulus-specific transcriptional responses, with a substantially higher number of deregulated genes detected in A549 cells exposed to MT-2 cells or their SN (Figure 2b, Supplementary Figure 1). Enrichment analysis of these DEGs identified pathways associated with antiviral defense, cytokine signaling, and NF-κB activation, which drew the selection of 105 candidate genes (HTLV-1 signature) for downstream analysis (Figure 2f).

HTLV-1-induced inflammation is characterized by an enhanced immune response within infected tissues. In the lung, this includes elevated numbers of T lymphocytes in bronchoalveolar lavage fluid (BALF) (Nakayama et al. 2013; Yamazato et al. 2003; Nakayama et al. 2011; Matsuyama et al. 2003) and high HTLV-1 PVL (Desgranges et al. 1989), both of which contribute to chronic inflammatory responses. Beyond acting as physical barriers, lung epithelial cells actively participate in immune surveillance, by producing cytokines and chemokines that may influence HAPD progression. In response to HTLV-1 exposure, A549 cells mounted a robust antiviral response, marked by the upregulation of interferon-stimulated genes (Figure 2f) *TNFSF14*, *ISG15*, *OAS3, CD80, CD86, TRIM22* and *BST2*, in agreement with our previous findings in HAM/TSP patients (Leal et al. 2018; Menezes et al. 2014). A key node in this interferon-driven response is STAT1, a central mediator of inflammatory signaling and antiviral responses (Meyts and Casanova 2021; Au-Yeung and Horvath 2018). By transducing interferon (IFN) signals, STAT1 regulates the expression of a broad array of antiviral and pro-inflammatory genes, thereby shaping the host immune response to HTLV-1. Notably, STAT1 dysregulation has been previously reported in HAM/TSP patients. Indeed, Tattermusch et al. measured elevated STAT1 protein levels in these patients and linked this to type I IFN signature (Tattermusch et al. 2012). Accordingly, our curated KEGG HTLV-1 gene signature revealed a *STAT1* upregulation across multiple datasets, both *in silico (*Figure 2f) and *in vivo* with patient-derived samples (Figures 5a, b, d, g). This parallel suggests that STAT1-driven inflammatory pathways observed in A549 cells may mirror mechanisms contributing to HTLV-1-associated lung pathology *in vivo*.

In addition to antiviral genes, A549 co-culture with MT-2 cells increased expression of pro-inflammatory cytokines (*TNF*, *IL6*, *CXCL8*, and *IL1A*), which reflects the heightened inflammatory state observed in HTLV-1-exposed A549 cells (Figure 2f, Supplementary Figures 5a–5d). These findings align with a previous *in vitro* study reporting increased production of pro-inflammatory cytokines and chemokines in HTLV-1-infected A549 cells (Teruya et al. 2008). Remarkably, elevated TNF has been associated with clinical worsening in HAM/TSP, while the systemic increase in IL-6 has been linked to inflammaging, a common phenomenon observed in HAM/TSP patients (Assone et al. 2022).

The observed HAPD-induced pro-inflammatory response seems, at least partially, mediated by NF-κB signaling. Indeed, HTLV-1-exposed A549 cells exhibited increased expression of NF-κB-related genes (*NFKB1*, *NFKB2*, *RELA*) (Figure 2f), consistent with the activation of pro-inflammatory and antiviral pathways. Among the NF-κB-regulated genes, *IL15* was particularly notable due to its strong association with both epithelial immune signaling (Holtzman et al. 2014) and the Th1-biased inflammatory response (Mishra et al. 2014) observed in HAM/TSP patients. *IL15* expression is *Tax*-dependent (Mariner et al. 2001) and tightly regulated by NF-κB (Figure 2f). IL-15 can be found at elevated levels in PBMCs of HAM/TSP patients. Blocking *IL15* expression can reduce PBMC proliferation (Azimi et al. 1999), underscoring its role in disease progression. In this study, the consistent upregulation of *IL15* across transcriptomic data sets (Figures 2f, 5a and d), as well as its identification in African-ancestry GWAS for HTLV-1 PVL (Figure 5g), further confirms its previously reported role as both a biomarker and therapeutic target in HAM/TSP (Enose-Akahata et al. 2008; Enose-Akahata et al. 2019).

Epithelial-driven pro-inflammatory signaling amplifies cytokines and chemokines production, which favors the recruitment of immune cells (e.g., monocytes) to the lungs. Macrophages are among the most abundant immune cells in the respiratory tract (Boorsma et al. 2013) and are essential for antiviral defense, controlling inflammation (Watanabe et al. 2019), and preserving tissue homeostasis (Dias et al. 2021; Wynn et al. 2013). Their versatility allows them to adopt either pro-inflammatory or anti-inflammatory phenotypes, depending on environmental cues (Tarique et al. 2015). During viral infection, monocytes migrate to inflamed sites in response to chemotactic signals (Shi and Pamer 2011) and differentiate into macrophages, which can either promote pathogen clearance or support tissue repair (Ingersoll et al. 2011).

In the present study, co-culturing A549 cells with HTLV-1–infected MT-2 cells induced a strong pro-inflammatory chemokine response (*CCL2*, *CCL5, CCL20*) and increased production of the local growth factor CSF-1 (Figure 3g). Chemotaxis assays with THP-1 cells confirmed a cell ratio- and time-dependent increase in monocyte migration toward A549 MT-2 SN (Figures 4a, 4c). THP-1-induced cell migration followed a typical Gaussian distribution, with an optimal chemokine concentration eliciting maximal migration. As of 24 h, the concentration of CCL2 present in the SN was likely supra-optimal, resulting in reduced THP-1 cell migration (Figure 4a). Comparison of different cell ratios also showed reduced chemotaxis at a CCL2 concentration of 100 ng/mL, which is most likely caused by receptor desensitization (Figure 4c). Beyond promoting monocyte recruitment, factors secreted into A549 MT-2 SN may also influence myeloid cell fate. Indeed, exposure of THP-1 cells or primary monocytes to MT-2 SN promoted their differentiation into macrophages (Figures 4f-4h), an effect that correlated with the increased mRNA levels of *CCL2* and *CSF1* observed in the A549 MT-2 co-culture (Figures 3d, 3g). Using a CRISPR/Cas9 RelA knockout cell line, we confirmed that *CSF1* expression following MT-2 co-culture was, at least partially, regulated by NF-κB signaling (Figure 3k), highlighting the pivotal effect of HTLV-1-driven NF-κB signaling. Consistently, complementary THP-1 differentiation assays using SN from A549 co-cultures (A549 control *versus* A549 p65KO) supported a CSF-1-associated mechanism underlying macrophage differentiation (Figure 4h).

In the context of HAPD, the presence of monocytes and differentiated macrophages in inflamed tissues can serve as a prognostic biomarker for pulmonary fibrosis (Kreuter et al. 2021; Planté-Bordeneuve et al. 2021). Indeed, elevated monocyte counts in the lung were previously associated with an increased risk of IPF progression, hospitalization or death (Kreuter et al. 2021; Zhang et al. 2022; Scott et al. 2019). On the other end, lung fibrogenesis has been shown to decrease significantly following depletion of circulating monocytes or when macrophage recruitment to the lung is blocked after injury in mouse models (Gibbons et al. 2011). The increased recruitment of monocytes during IPF development was recently linked to age-associated changes. Farhat *et al.* (2025) demonstrated that an aged hematopoietic system can enhance the risk of lung fibrosis in young mice (Farhat et al. 2025). This effect was associated with an increased influx of monocytes, which gave rise to profibrotic macrophages in lung tissue (Farhat et al. 2025). In this study, STRING analysis of our curated KEGG gene list identified different hub proteins, including tissue monocyte markers as well as bronchiectasis-associated proteins (Figure 3a). Together, these findings underscore the capacity of HTLV-1-exposed epithelial cells to influence, not only monocyte recruitment but also the functional activation of myeloid cells in the lung and its contribution to IPF development. All these results aligned with findings from a recently published study, in which the authors confirmed the role of secreted factors of HTLV-1-infected cells in monocyte activation and differentiation (Souza et al. 2025).

To explore the *in vivo* relevance of our *in silico* and *in vitro* findings, we refined an HTLV-1 infection signature by cross-referencing our expression data with published whole blood transcriptomic profiles from HAM/TSP patients (Tattermusch et al. 2012) (Figures 5a-b). Genes upregulated in A549 MT-2 co-cultures also showed a strong overlap with a curated IPF gene list (Figures 5c-e) and multi-ancestry GWAS data (Figures 5f, g, Supplementary Figure 7). In a recent preprint, Assone *et al*. used systems biology analyses of novel and publicly available data comprising (epi)genomics, transcriptomics, metabolomics and proteomics of multi-ancestry cohorts from a total of >2500 people living with HTLV-1 from 5 countries (Brazil, Peru, Japan, UK, US) (Van Weyenbergh et al. 2025). In a unique admixed Brazilian cohort, GWAS revealed both general and ancestry-specific genetic polymorphisms. Systems biology analysis revealed neuronal/synaptic signaling, monocyte count, glucose/lipid metabolism, and neurocognition/depression, as genetically linked to HAM/TSP patients, for which higher monocyte levels were validated in independent Brazilian and Peruvian cohorts (Van Weyenbergh et al. 2025). Similar to our findings in HTLV-1-exposed A549 cells, Assone *et al.* found strong biological similarities between retroviral Hbz/Tax overexpression and HAM multi-omics findings, including viral pathways such as "Epstein-Barr virus", recently identified as the major driver of multiple sclerosis (Van Weyenbergh et al. 2025). Finally, we compared our filtered KEGG gene list (Figure 2f) with single-cell datasets of lung tissues from the HLCA (Sikkema et al. 2023), which revealed a specific CCL2-high myeloid cell subset (Figure 5h, Supplementary Figure 8). Notably, this subset was strongly correlated with the previously defined HAM/TSP type I IFN gene signature (Tattermusch et al. 2012), indicating that these cells may contribute to the inflammatory responses observed in HTLV-1-exposed A549 cells.

The present study has three major limitations. First, confirming infection of A549 cells exposed to HTLV-1-infected cells or their SN is technically challenging. HTLV-1 primarily spreads via cell-to-cell contact through virological synapses, biofilm-like structures and cellular conduits, as well as through tunneling nanotubes (Nejmeddine et al. 2009; Omsland et al. 2018). Although the *in vitro* coculture model recapitulates *in vivo* clinical findings in people living with HTLV-1(Figures 5a-5g), we did not demonstrate HTLV-1 infection in A549 cells, which was reported previously (Teruya et al. 2008). In addition, recent *in vivo* work has confirmed infection of lung tissue in HTLV-1-infected macaques, underscoring the importance of pulmonary involvement in HTLV-1 pathogenesis (Sarkis et al. 2025). Interestingly, our DEG analysis revealed significant transcriptional changes in A549 cells exposed to MT-2 SN compared to A549 control or A549 cells exposed to MT-4 SN (Figure 2b). Notably, A549 cells co-cultured with MT-2 cells or their SN shared a large proportion of DEGs, with over 70 of our 105 selected genes differentially expressed under both conditions (Figure 2f). This overlap suggests that, although residual MT-2 cell carryover in co-culture cannot be fully excluded, the observed biological effects are largely driven by HTLV-1 components present in the SN and remain biologically meaningful. Despite the rarity of *in vitro* HTLV-1 infection in A549 cells and the potential presence of residual MT-2 cells, HTLV-1 exposure induced marked transcriptomic reprogramming in A549 cells, consistent with *in vivo* findings in lung tissues (Figure 5). The second limitation consists of the use of A549 adenocarcinoma cells instead of primary human lung cells. Nevertheless, similar to our findings, A549 cells have been shown to recapitulate transcriptomic findings of Zika virus-infected patients upon *in vitro* infection, and were used to study immune-epithelial cell cross-talk in SARS-CoV-2 infection (Berglund 2024; Magalhães et al. 2023). Moreover, CRISPR genome editing has not been achieved yet in primary human lung cells, to the best of our knowledge. The third limitation lies in the absence of *in vivo* or single-cell data from HTLV-1-infected lung tissues. HTLV-1 is still a highly neglected virus, with limiting access to clinically relevant samples. Obtaining such datasets is also technically challenging due to the invasive nature of lung biopsies and the difficulty of securing ethical approval, which hampers the establishment of large public biobanks. Recently, however, a study in HTLV-1-infected macaques confirmed HTLV-1 involvement in the respiratory system (Sarkis et al. 2025). Notably, all macaques infected with HTLV-1A cloned with the *Orf I* of the HTLV-1C strain developed bronchiectasis within 10 months of infection. The authors reported elevated IL-6, CCL2, and IL-1β levels in the lung. In addition, bronchoalveolar lavage (BAL) samples from the HTLV-1A-infected subgroup showed increased IL-15 and IL-1β, which were associated with higher frequencies of classical and non-classical monocytes producing IL-10 in blood (Sarkis et al. 2025), corroborating our *in vitro* (this study) and *in vivo (*Assone et al. 2024) findings. Together, these findings demonstrate *in vivo* HTLV-1 infection in the lung and its strong association with bronchiectasis development in HAPD.

Despite these limitations, the main strength of this study lies in its multi-omics design (Figure 1). By integrating *in vitro* data and systems biology analysis, we were able to extend our findings to the *in vivo* level,combining multi-omics data from several independent cohorts worldwide, including healthy controls, people living with HTLV-1 and HAM/TSP patients.

## Conclusions

**Fig. 6:**
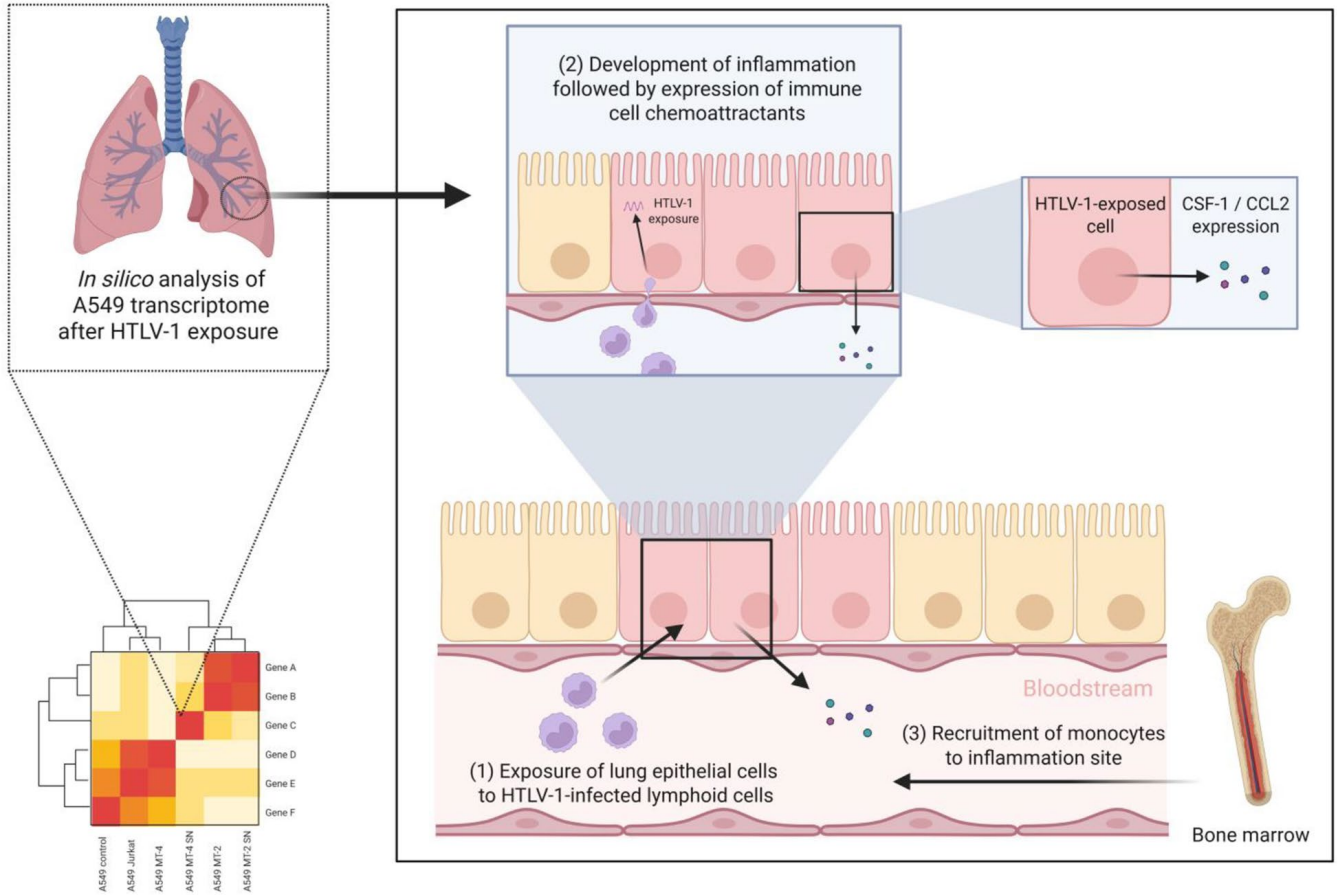
HTLV-1 exposure induces monocyte recruitment to the inflamed lung. Using a multi-omics framework, we demonstrated that HTLV-1 exposure induces acute inflammation in A549 alveolar epithelial cells. The resulting pro-inflammatory response promotes immune cell recruitment via the CSF-1/CSF-1R axis

Using an integrated multi-omics framework, our data-driven approach uncovers novel disease mechanisms and potential therapeutic targets for HTLV-1-associated lung pathology (Figure 6). Systems biology analysis showed RelA/NF-κB p65 as the major upstream transcription factor for lung-specific HTLV-1-upregulated genes. A central role for CSF-1-mediated recruitment and differentiation of monocytes was mechanistically linked to NF-κB activation, as demonstrated using a CRISPR/Cas9 A549 RelA knockout cell line. A strong molecular overlap with both HAM/TSP and IPF reveals shared immunopathogenic pathways between unrelated pathologies targeting the lung. Together, these experimental and transcriptomic data support a model in which HTLV-1 drives chronic alveolar inflammation via epithelial-derived cytokine release and monocyte recruitment, while subsequent differentiation into inflammatory/profibrotic macrophages may contribute to viral persistence, immune dysregulation, and progression toward fibrotic lung disease. Integrated multi-cohort multi-omics analyses, including bulk and single-cell transcriptomics, viral interactome data, and cross-ancestry GWAS, provide support for the *in vivo* relevance of these pathways. Building on these findings, further *in vivo* studies will enable direct testing of the proposed mechanism, strengthening the translational relevance of our study. More broadly, this work demonstrates the use of an integrated multi-omics framework to investigate inflammatory disease mechanisms in a novel *in vitro* co-culture system allowing molecular mechanistic studies such as CRISPR/Cas9 genome editing, which are currently not possible in humans.

## Supplementary Information

Supplementary Material 1. Supplementary Figure 1. Transcriptomic analysis of A549 co-culture with lymphoids cells – complementary data. Supplementary Figure 2. Cross-comparison of in vitro transcriptomic data with publicly available single-cell lung epithelial datasets. Supplementary Figure 3. Transcriptomic analysis of A549 cells co-cultured with HTLV-1-infected MT-2 cells reveals upregulation of genes involved in antiviral signaling, inflammatory response, and NF-κB activation. Supplementary Figure 4. Transcriptomic analysis of A549 cells co-cultured with HTLV-1-infected MT-2 cells reveals upregulation of genes involved in cell chemotaxis and differentiation. Supplementary Figure 5. Exposure of lung epithelial cells to HTLV-1-infected lymphocytes induces inflammatory signaling and NF-κB–dependent responses. Supplementary Figure 6.ex vivo HOST cohort. Supplementary Figure 7. Transcriptomic overlap with HAM/TSP GWAS data. Supplementary Figure 8. Characterization of the 105-gene HTLV-1 signature in the Human Cell Atlas lung dataset.

Supplementary Material 2. Supplementary Table 1. Overview raw counts RNAseq analysis. Supplementary Table 2. Transcriptome A549 Jurkat co-culture. Supplementary Table 3. Transcriptome A549 MT-4 co-culture. Supplementary Table 4. Transcriptome A549 MT-4 SN co-culture. Supplementary Table 5. Transcriptome A549 MT-2 co-culture. Supplementary Table 6. Transcriptome A549 MT-2 SN co-culture. Supplementary Table 7. Upstream Transcription Factor enrichment analysis performed on the 105 selected filtered KEGG genes. Supplementary Table 8. Upstream Transcription Factor enrichment analysis performed on the 105 selected filtered KEGG genes. Supplementary Table 9. Top 20 Most significant upregulated genes in A549 cell co-cultured with MT-2/MT-2 SN or MT-4/MT-4 SN. Supplementary Table 10. KEGG enrichment analysis performed on 830 upregulated genes in A549 MT-2 co-culture. Supplementary Table 11. Gene Ontology enrichment analysis performed on 830 upregulated genes in A549 MT-2 co-culture.

## Data Availability

Transcriptomic data generated in this study were submitted to the National Institutes of Health (NIH) and can be found upon release under the following GSE number: **GSE312068**. All materials generated in this study will be provided upon request.
