# Supplementary material for: Integrated in vitro and multi-cohort cross-omics analysis of HTLV-1-associated lung pathology reveals a RelA-dependent mechanism for monocyte recruitment and differentiation
Source: Mol Med. 2026 Apr 23;32:85. doi: 10.1186/s10020-026-01482-9 (PMC13237950; doi:10.1186/s10020-026-01482-9)
Supplement: Supplementary file 1 — Supplementary Material 1. Supplementary Figure 1. Transcriptomic analysis of A549 co-culture with lymphoids cells – complementary data. Supplementary Figure 2. Cross-comparison of in vitro transcriptomic data with publicly available single-cell lung epithelial datasets. Supplementary Figure 3. Transcriptomic analysis of A549 cells co-cultured with HTLV-1-infected MT-2 cells reveals upregulation of genes involved in antiviral signaling, inflammatory response, and NF-κB activation. Supplementary Figure 4. Transcriptomic analysis of A549 cells co-cultured with HTLV-1-infected MT-2 cells reveals upregulation of genes involved in cell chemotaxis and differentiation. Supplementary Figure 5. Exposure of lung epithelial cells to HTLV-1-infected lymphocytes induces inflammatory signaling and NF-κB–dependent responses. Supplementary Figure 6.ex vivo HOST cohort. Supplementary Figure 7. Transcriptomic overlap with HAM/TSP GWAS data. Supplementary Figure 8. Characterization of the 105-gene HTLV-1 signature in the Human Cell Atlas lung dataset. [file 10020_2026_1482_MOESM1_ESM.pdf]

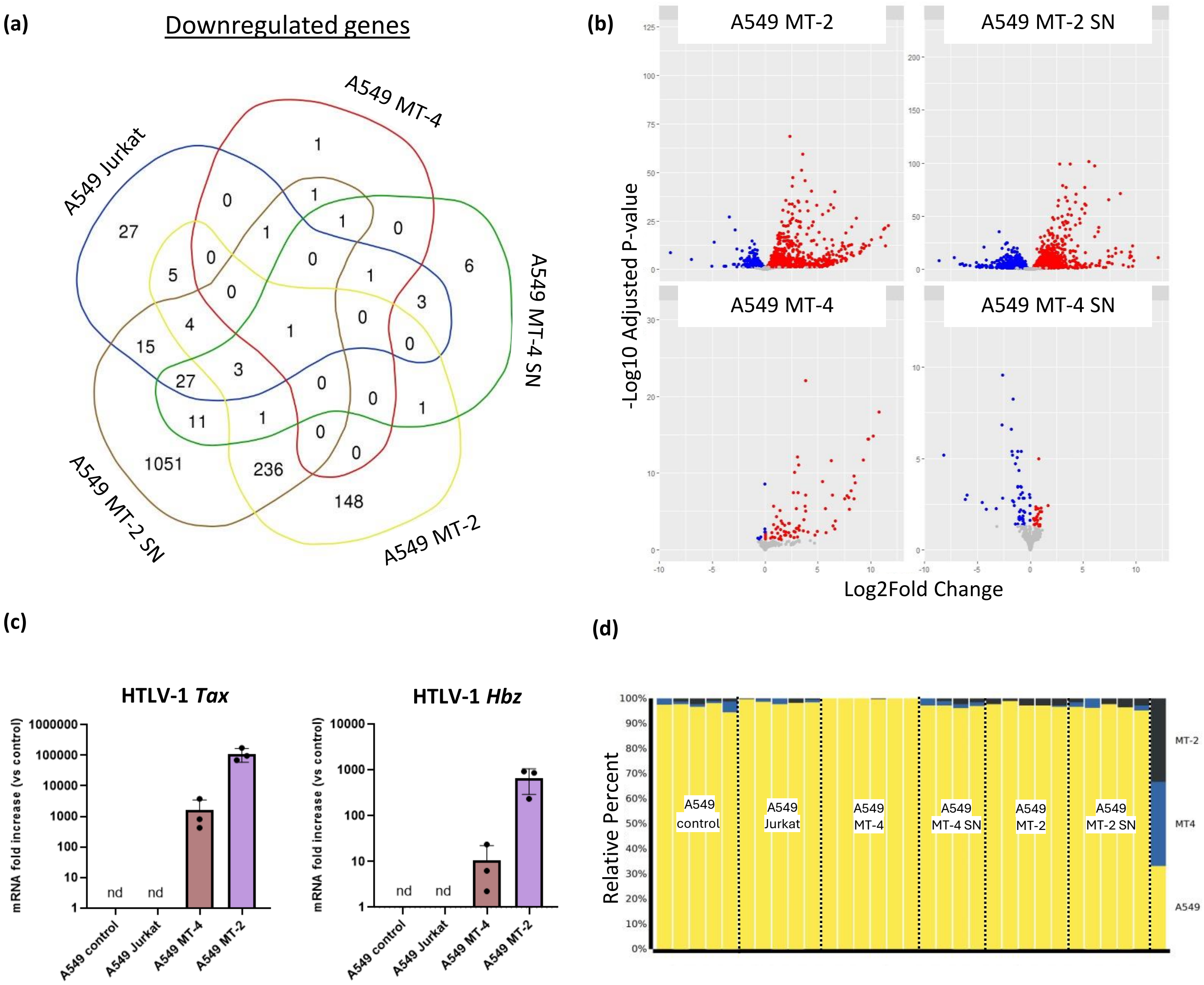

Supplementary Figure 1. Transcriptomic analysis of A549 co-culture with HTLV-1-infected or uninfected lymphoids cells – complementary data. A total of 4x10^5 A549 lung epithelial cells were co-cultured with Jurkat, MT-4 (or SN), or MT-2 (or SN) cells at a 1:1 ratio for 24 hours (n = 4-6 per condition). RNA was isolated, followed by bulk RNAseq (see Figure 1). **(a)** Venn diagram of downregulated DEGs illustrate overlapping and unique gene expression profiles; A549 MT-2 and MT-2 SN co-cultures exhibited largely distinct signatures, compared to A549 Jurkat and A549 MT-4 conditions. **(b)** Volcano plots show DEGs identified in the different A549 co-cultures (MT-2 and MT-4 conditions), using A549 Jurkat as control reference. **(c)** RT-qPCR detection of HTLV-1 *Tax* and *Hbz* mRNA in A549 cells after 48 h of MT-2 co-culture (n = 3) suggests indirect viral transmission. HTLV-1 *Tax* and *Hbz* mRNA were undetectable in A549 control and A549 Jurkat co-cultures (= nd). Hence, a Ct value of Ct 35 was attributed by default to all these samples. Fold changes were calculated, using A549 control as reference. **(d)** Deconvolution analysis of bulk RNA sequencing from A549 co-cultures. Normalized reads from A549 co-cultures were compared to normalized reads from A549 control (RNA-seq) and from MT-2 and MT-4 cells (nCounter data). Using CIBERSORTx, reference expression profiles from each monoculture were used to estimate the relative proportions of A549, MT-2, and MT-4 cells in co-cultures.

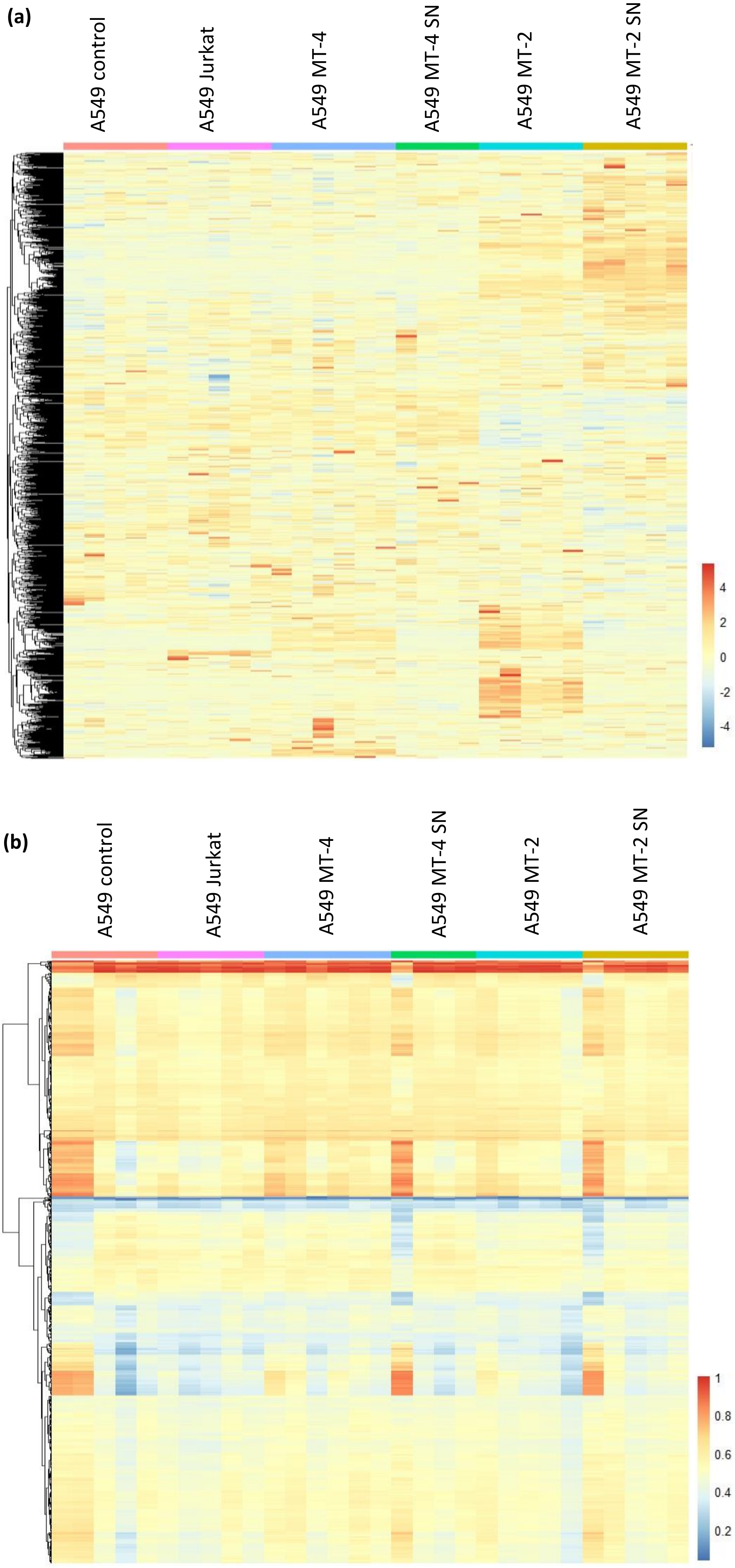

Supplementary Figure 2. Cross-comparison of *in vitro* transcriptomic data with publicly available single-cell lung epithelial datasets. To better understand the impact of HTLV-1 infection on lung epithelium, 4×10⁵ A549 lung epithelial cells were co-cultured with HTLV-1-infected or uninfected lymphoid cells and subjected to RNA sequencing. **(a)** The resulting gene expression data were compared to a curated list of epithelial markers obtained from the PanglaoDB database. Fold changes overlapping between both datasets were plotted in the heatmap. **(b)** To complement the first heatmap, a correlation analysis was performed by comparing raw RNA-seq counts from this study with aggregated read counts from A549 RNA-seq experiments available in the ARCHS4 database. Only genes expressed in more than three samples with a sequencing depth exceeding 200 reads were retained for analysis.

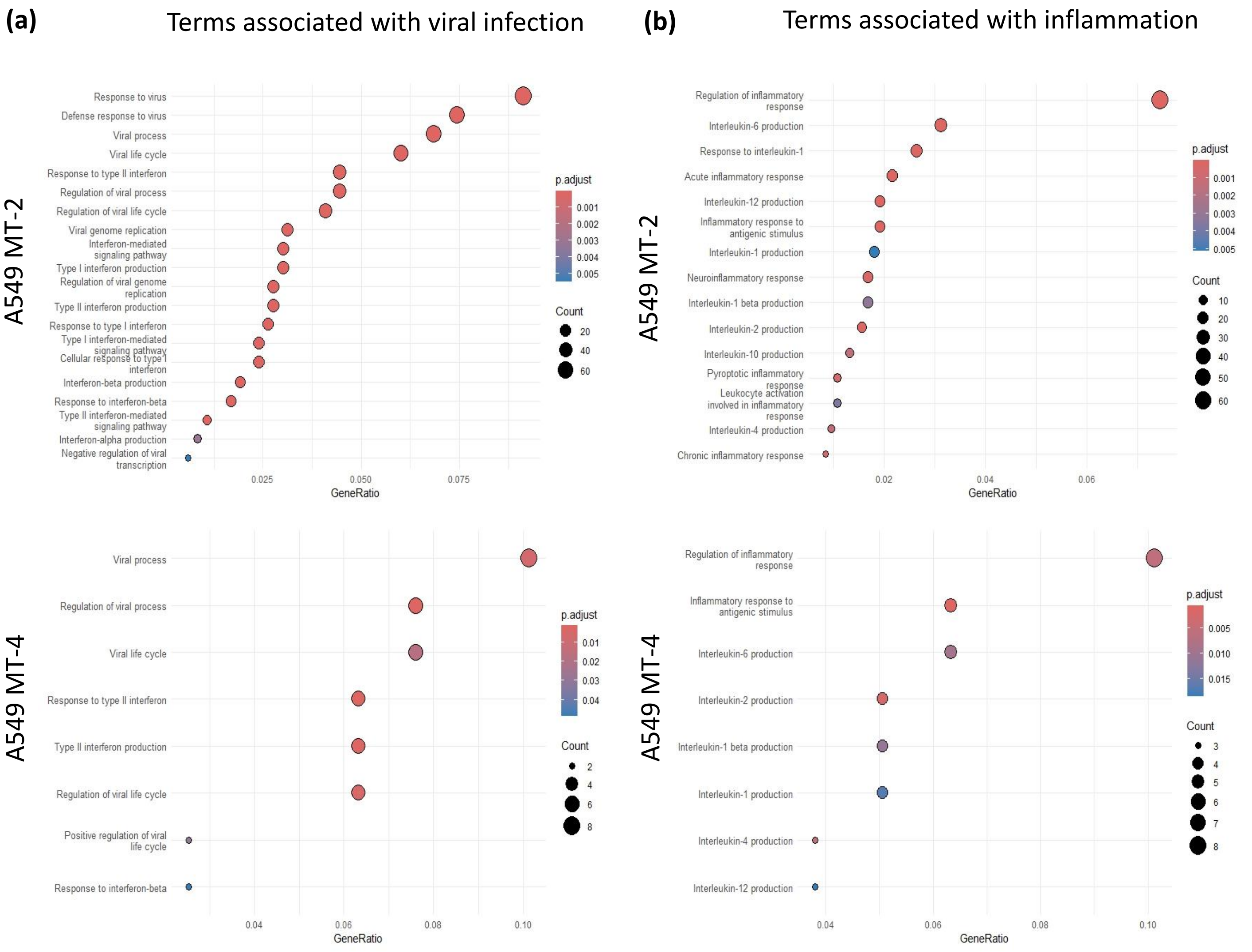

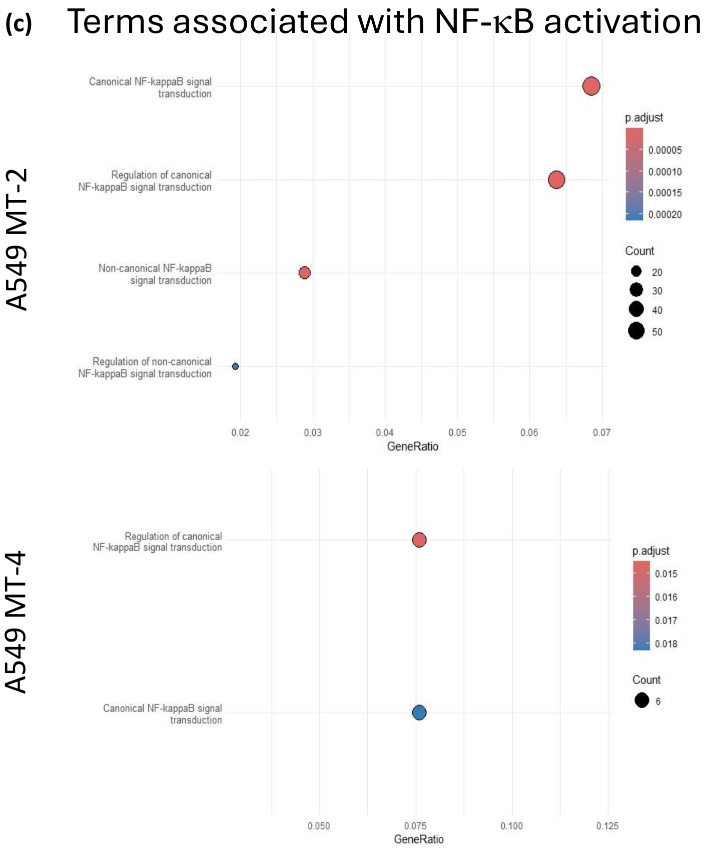

Supplementary Figure 3. Transcriptomic analysis of A549 cells co-cultured with HTLV-1-infected MT-2 cells revealed upregulation of genes involved in antiviral signaling, inflammatory response, and NF-κB activation. Gene Ontology (GO) enrichment analysis was performed on upregulated DEGs identified from A549 cells co-cultured with HTLV-1-infected MT-2 or MT-4 cells. Significantly enriched GO terms were selected based on clinical relevance. **(a)** Co-culture with MT-2 cells led to the upregulation of genes associated with viral infection. **(b)** MT-2 exposure also induced an inflammatory response in A549 cells, as indicated by enrichment of pro-inflammatory pathways. **(c)** HTLV-1 exposure activated NF-κB signaling in A549 cells co-cultured with MT-2 cells.

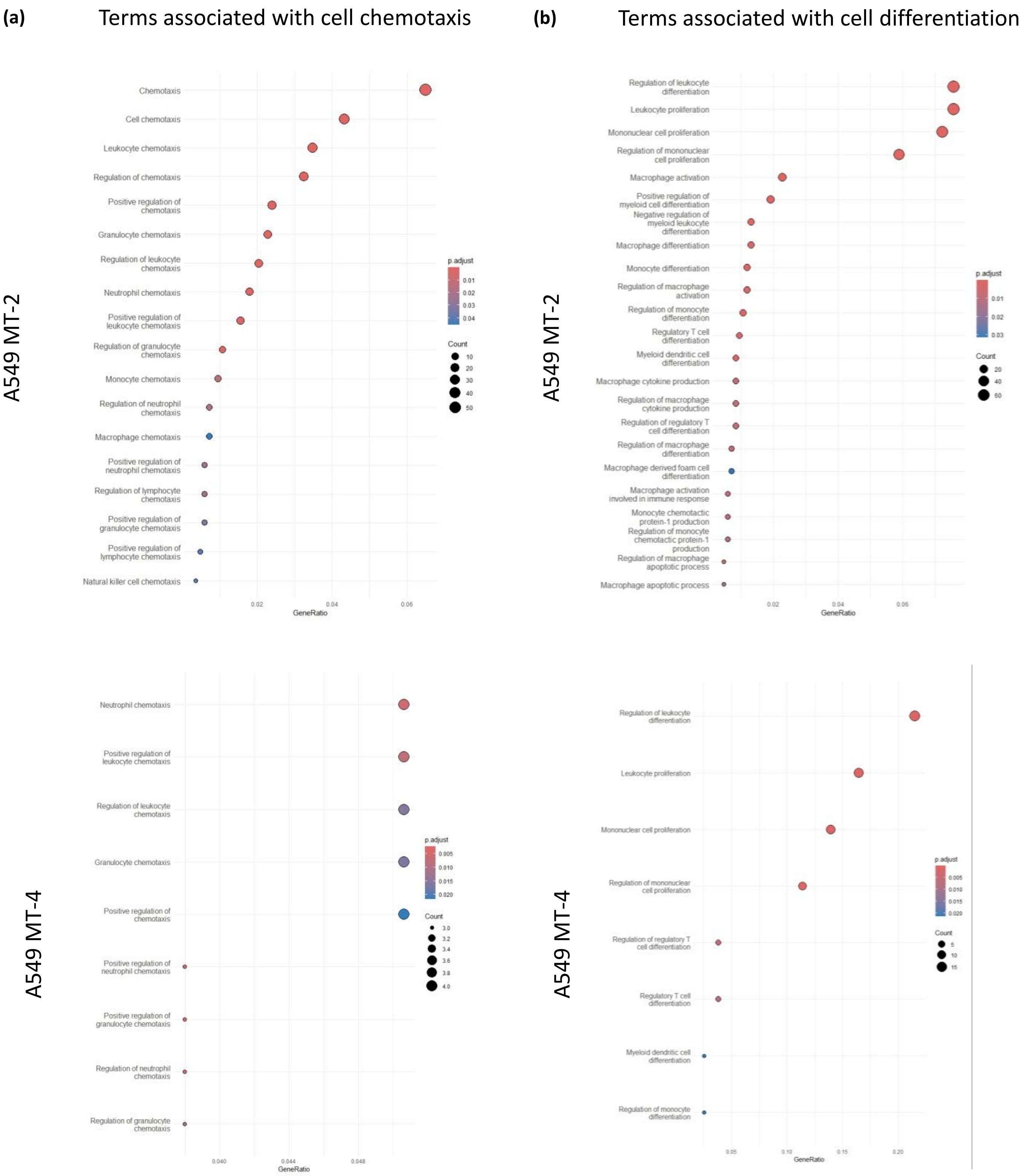

Supplementary Figure 4. Transcriptomic analysis of A549 cells co-cultured with HTLV-1-infected MT-2 cells revealed upregulation of genes involved in cell chemotaxis and differentiation. Gene Ontology (GO) enrichment analysis was performed on upregulated differentially expressed genes (DEGs) from A549 cells co-cultured with HTLV-1-infected MT-2 or MT-4 cells. Clinically relevant GO terms with significant enrichment were selected for further interpretation. **(a)** Co-culture with MT-2 cells led to the upregulation of genes involved in the regulation of the host immune response, as indicated by enrichment of GO terms related to cell chemotaxis. **(b)** HTLV-1 exposure also induced the activation of pathways associated with cell differentiation in A549 cells.

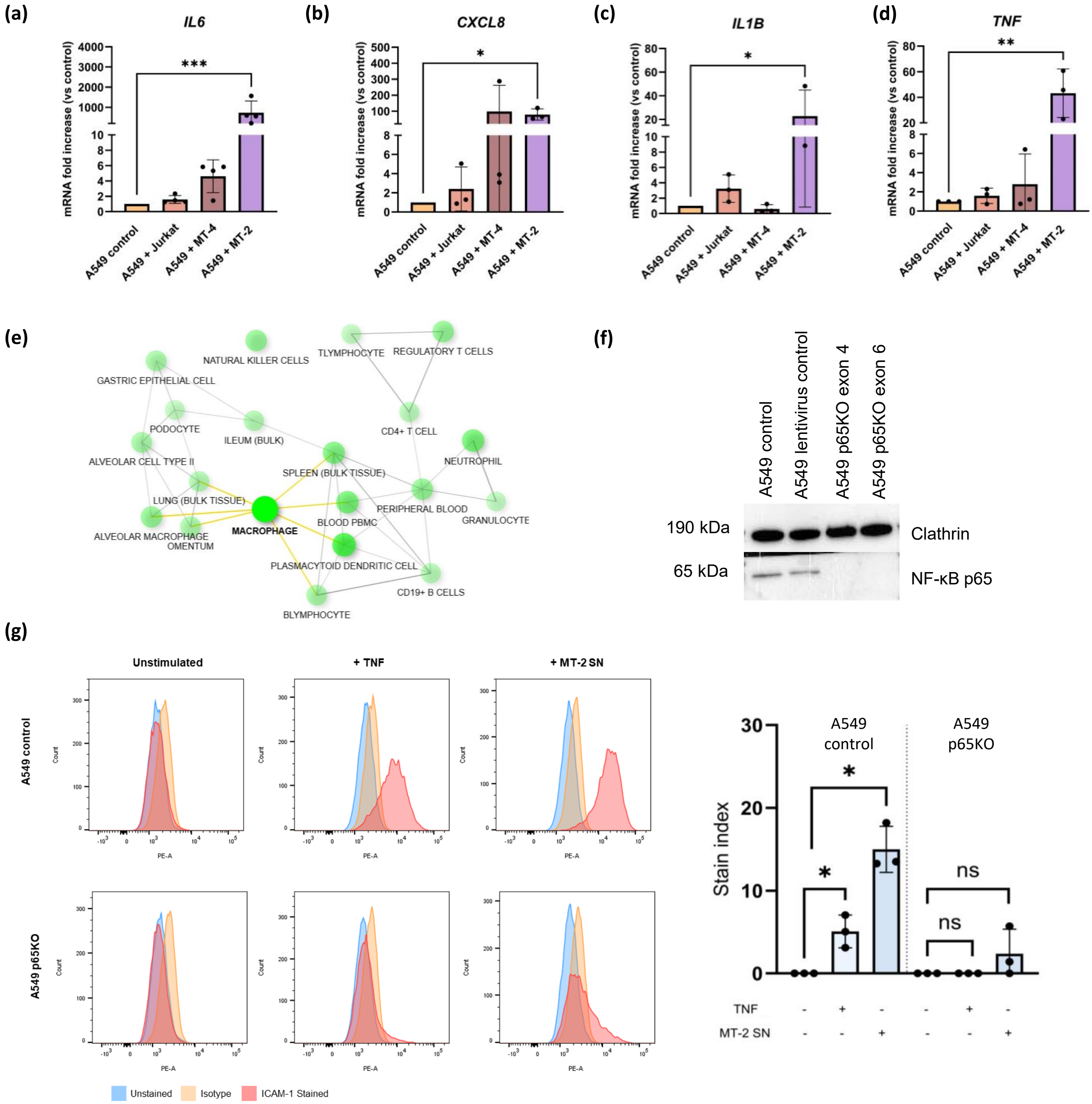

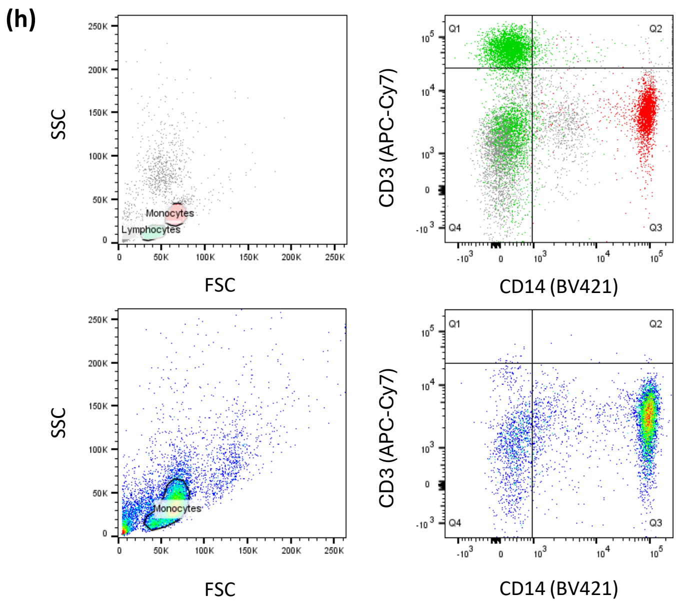

Supplementary Figure 5. Exposure of lung epithelial cells to HTLV-1–infected lymphocytes induced inflammatory signaling and NF-κB–dependent responses. A total of 4×10^5 A549 cells were co-cultured with HTLV-1-infected (MT-2 or MT-4) or uninfected (Jurkat) lymphoid cells for 48 hours. Total RNA was extracted and analyzed by RT-qPCR. **(a–d)** A549 cells co-cultured with MT-2 cells showed significantly elevated expression of pro-inflammatory cytokines compared to A549 control. **(e)** Comparison of the 105 genes selected after KEGG enrichment analysis (see Figure 2) with the ARCHS4 Tissue database indicated that these genes are predominantly regulated by macrophage-associated transcription factors. **(f)** Western blot confirmed CRISPR-Cas9-induced NF-κB p65 (RelA) knockout in A549 cells. **(g)** Confirmation of NF-κB p65 knockout in A549 cells by downstream target ICAM-1, as quantified by flow cytometry. A549 cells were stimulated with pro-inflammatory cytokine TNF (50 ng/mL) or MT-2 SN for 6 h and ICAM-1 expression on the cell surface was measured. **(h)** Primary CD14$^+$ monocytes were isolated from healthy donors via density gradient and magnetic sorting, with purity validated by flow cytometry. Statistical significance was determined by non-parametric Kruskal-Wallis ANOVA, followed by a Tukey Post Hoc Test. *$p < 0.05$; **$p < 0.01$; ***$p < 0.001$; ****$p < 0.0001$.

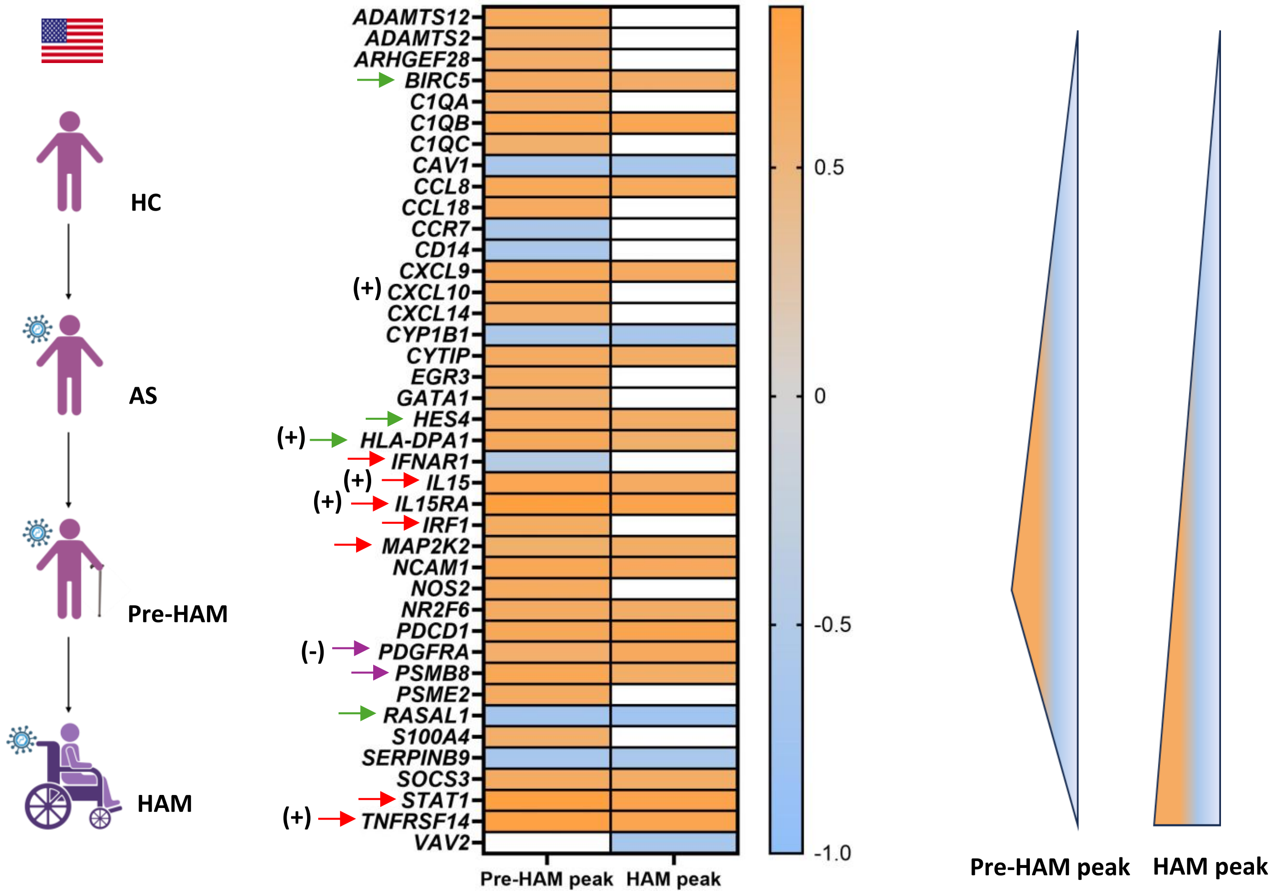

Supplementary Figure 6. *Ex vivo* HOST cohort. *Ex vivo* transcriptomic data from HOST cohort identified genes associated with HTLV-1 clinical status and/or disease burst. (Purple: IPF downregulated; Green: IPF upregulated; Red: Filtered KEGG gene list; (–/+): Genes negatively or positively regulated by HTLV-1 Tax). Statistical significance: padj < 0.05.

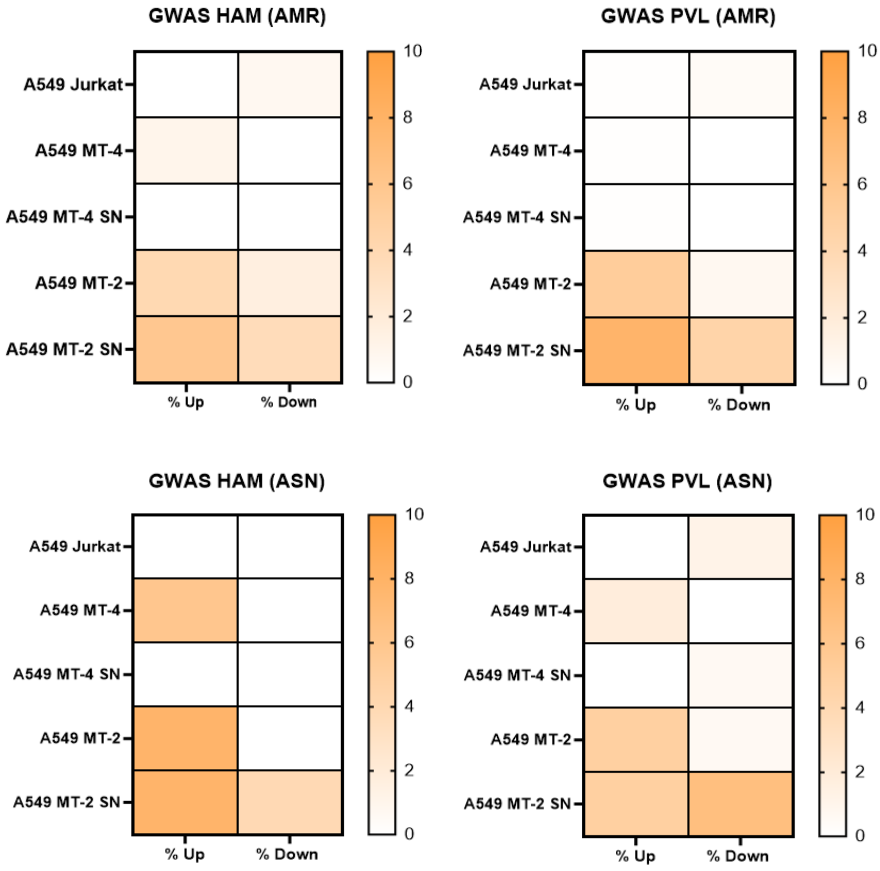

Supplementary Figure 7. Transcriptomic overlap with HAM/TSP GWAS data. Following differential gene expression analysis on A549 co-cultures, obtained DEGs were compared with data from multi-ancestral GWAS study. Heatmaps illustrate overlap percentages between A549-derived DEGs and transcripts from the different GWAS datasets (HAM and HTLV-1 PVL; Amerindian (AMR) and Asian (ASN) ancestry).

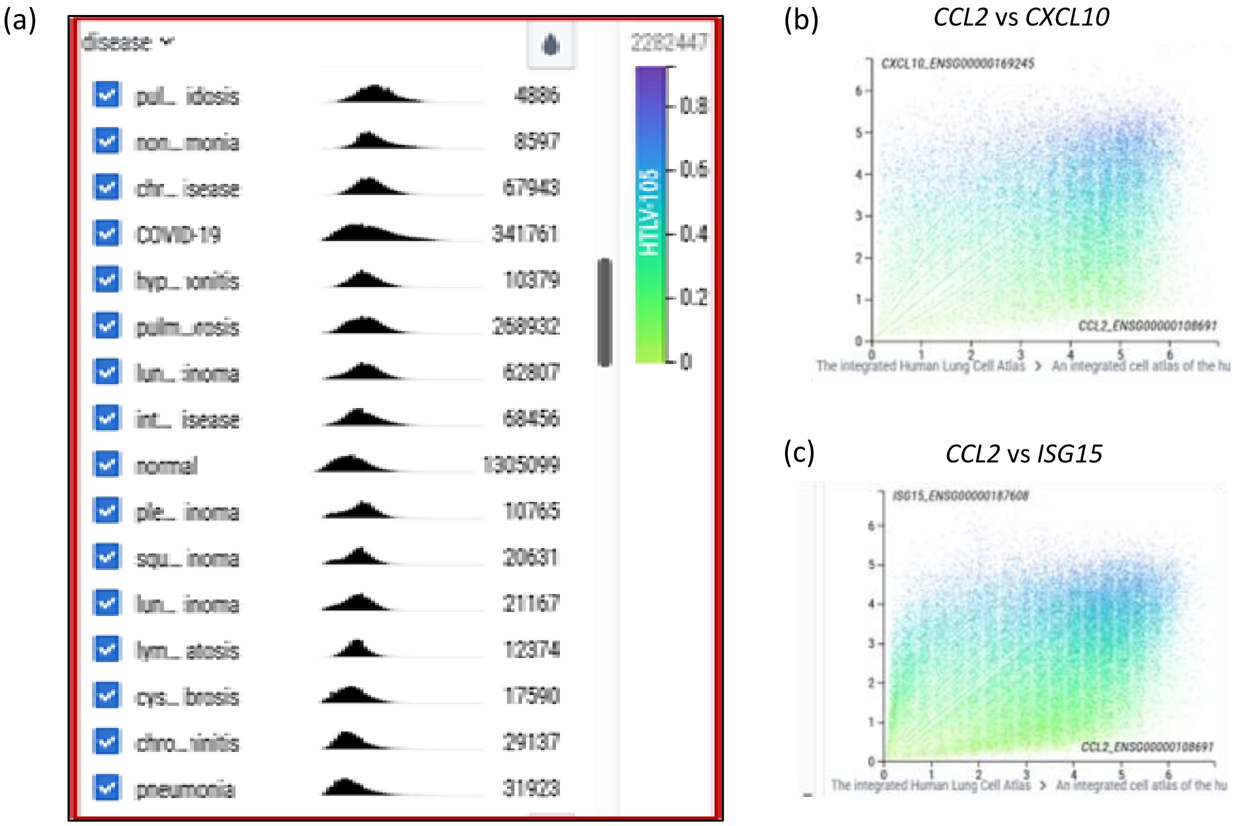

Supplementary Figure 8. Characterization of the 105-gene HTLV-1 signature in the Human Cell Atlas lung dataset. Comparative analysis between our proposed HTLV-1 gene signature and single-cell RNA sequencing data from patients with various inflammatory lung diseases identified a distinct CCL2^{+} myeloid cell subset. **(a)** Expression of the 105-gene HTLV-1 signature was elevated across multiple inflammatory lung conditions, validating the clinical and pathological relevance of the in vitro-derived model. **(b-c)** Within lung tissue, *CCL2* expression showed strong positive correlation with *ISG15* and *CXCL10*, two hallmark markers of the HAM/TSP-associated interferon (IFN) gene signature, highlighting a shared inflammatory axis between HTLV-1-driven neuroinflammation and pulmonary immune activation.
